# A previously uncharacterized gene, PA2146, contributes to biofilm formation and drug tolerance across the ɣ-Proteobacteria

**DOI:** 10.1038/s41522-022-00314-y

**Published:** 2022-07-07

**Authors:** Matthew F. Kaleta, Olga E. Petrova, Claudia Zampaloni, Fernando Garcia-Alcalde, Matthew Parker, Karin Sauer

**Affiliations:** 1 Department of Biological Sciences, Binghamton University, Binghamton, NY 13902 USA; 2 Binghamton Biofilm Research Center, Binghamton University, Binghamton, NY 13902 USA; 3 Roche Pharma Research and Early Development, Immunology, Inflammation and Infectious Diseases, Roche Innovation Center Basel, F. Hoffmann-La Roche Ltd, Grenzacherstrasse 124, 4070 Basel, Switzerland

**Keywords:** Biofilms, Molecular evolution

## Abstract

Transcriptomic studies have revealed a large number of uncharacterized genes that are differentially expressed in biofilms, which may be important in regulating biofilm phenotypes such as resistance to antimicrobial agents. To identify biofilm genes of unknown function in *P. aeruginosa*, we made use of RNA-seq and selected 27 uncharacterized genes that were induced upon biofilm growth. Biofilms by respective mutants were subsequently analyzed for two biofilm characteristics, the biofilm architecture and drug susceptibility. The screen revealed 12 out of 27 genes to contribute to biofilm formation and 13 drug susceptibility, with 8 genes affecting both biofilm phenotypes. Amongst the genes affecting both biofilm phenotypes was PA2146, encoding a small hypothetical protein that exhibited some of the most substantial increases in transcript abundance during biofilm growth by *P. aeruginosa* PAO1 and clinical isolates. PA2146 is highly conserved in ɣ-proteobacteria. Inactivation of PA2146 affected both biofilm phenotypes in *P. aeruginosa* PAO1, with inactivation of homologs in *Klebsiella pneumoniae* and *Escherichia coli* having similar effects. Heterologous expression of PA2146 homologs complemented the *P. aeruginosa* ∆PA2146, suggesting that PA2146 homologs substitute for and play a similar role as PA2146 in *P. aeruginosa*.

## Introduction

Bacteria preferentially grow as biofilm communities in diverse settings including the natural environment, industrial systems, and the medical sphere^1–3^. Growth within biofilms offers protection from adverse conditions, such as defense from protozoan grazing in the marine environments, resistance to antimicrobial agents during decontamination of industrial and medical equipment, and evasion of host immune responses during infections^4–7^. Evidence of this protected mode of growth appears early in the fossil record (~3.25 billion years ago) and is common throughout a diverse range of organisms in both the Archaea and Bacteria lineages, suggesting biofilm growth to be an integral component of the prokaryotic life cycle^8^. Indeed, studies of biofilms formed by diverse prokaryotes have revealed common trends and phenotypic characteristics of biofilms, as addressed by several reviews^9–12^. These common trends include cell-to-cell communication or quorum sensing (QS), the production of extracellular polymeric substances to form a protective matrix, the presence of eDNA as a matrix component, and the modulation of motility, adhesins, and c-di-GMP levels having been identified as key factors in the formation of biofilms. Additionally, the upregulation of genes involved in adaptation to stationary phase, environmental stress, and anaerobiosis have been identified as common features associated with biofilm growth^13,14^. Taken together, these findings led to the notion of biofilm cells being distinct from stationary-phase cells and having certain traits that are more common in biofilm-associated than in planktonic cells.

Transcriptomic studies also revealed large numbers of uncharacterized genes/proteins being induced upon biofilm growth. In fact, hypothetical and conserved hypothetical genes constituted the largest category of differentially regulated genes. Using RNA-seq, Iraola et al.^15^ found 289 out of 575 (50%) genes to be differentially expressed under conditions associated with biofilm growth by *Leptospira biflexa* that were annotated as hypothetical protein-encoding genes. Whiteley et al.^16^ determined that biofilm formation by *P. aeruginosa* coincided with about 34% of the 73 biofilm-regulated genes coded for hypothetical proteins of unknown function. Moreover, exposure of biofilms to tobramycin coincided with the differential expression of 20 genes, 12 of which were classified as genes coding for hypothetical proteins of unknown function^16^. Similarly, a transcriptomic analysis carried out by Seneviratne et al.^17^ indicated *Enterococcus faecalis* biofilm formation and resistance to antimicrobial agents including arsenic, to coincide with the differential expression of a large number of putative genes. In an effort to determine genes correlated with the surface‐associated mode of growth by *Vibrio cholerae*, Moorthy and Watnick^18^ identified 12 clusters of genes encoding proteins of unknown function that demonstrated similar expression patterns under surface-associated conditions including growth as monolayers or biofilms, compared to the planktonic mode of growth.

The large number of uncharacterized genes/proteins that are differentially expressed in biofilms may not come as a surprise considering that a large portion of sequenced bacterial genomes (25–40%) encode hypothetical genes or genes of unknown function. However, the findings may also suggest that such factors may play important roles for the biofilm mode of growth. In *E. faecalis*, many of the uncharacterized loci that artbe expressed during biofilm formation^17,19–21^, have likewise been detected in animal models of infection^22,23^, as well as following exposure to antibiotics^24^. Similar findings have been reported in other bacterial species such as *P. aeruginosa*^25,26^. Given the prevalence and reoccurring detection of hypothetical and uncharacterized genes/proteins under in vitro and in vivo biofilm-related conditions, it stands to reason that we may have missed factors or pathways that contribute to biofilm formation. Therefore, the goal of this study was to determine if hypothetical and previously uncharacterized genetic determinants contribute to biofilm formation, by using the biofilm model organism *Pseudomonas aeruginosa* and focusing on two biofilm characteristics, the biofilm architecture and biofilm antimicrobial tolerance.

## Results

### Genes encoding previously uncharacterized proteins contribute to the formation of biofilms by *P. aeruginosa*

To uncover hypothetical and previously uncharacterized factors associated with biofilm formation and/or biofilm antimicrobial tolerance in *P. aeruginosa*, we first assessed the transcriptomes of *P. aeruginosa* PAO1 cells grown planktonically and as biofilms using RNA-seq. Under the conditions tested, a total of 1812 genes (or approximately a third of the genome) were found to be differentially expressed in biofilms. Of these, genes encoding previously uncharacterized (hypothetical) proteins (Supplementary Table 1) accounted for more than a third of the biofilm-specific transcriptomic changes in *P. aeruginosa*. Moreover, many of the genes encoding previously uncharacterized proteins have not been previously linked to biofilm developmental processes, underscoring our still limited understanding of biofilm formation. A subset of the genes that were found to be differentially expressed by RNA-seq were subjected to qRT-PCR analysis to confirm differential transcript abundance upon biofilm growth (Table 1, Supplementary Table 2).

**Table 1:** Qualitative analysis of biofilm related phenotypes and transcript abundance of genes of interest.

Strain	Biofilm architecture^a,b^	Susceptibility phenotype^a,c,d^		PA number	Relative transcript abundance in biofilms	
		Tobramycin	Hydrogen Peroxide		RNA-seq	qRT-PCR
**PAO1**	WT-like	WT-like	WT-like			
**PA0452::IS**	WT-like	WT-like	WT-like	**PA0452**	9.0974175	5.26
**PA2116::IS**	WT-like	WT-like	WT-like	**PA2116**	4.4021755	n.d.
**PA2134::IS**	WT-like	WT-like	WT-like	**PA2134**	3.3907125	n.d.
**PA2377::IS**	WT-like	WT-like	WT-like	**PA2377**	18.138506	n.d.
**PA2508::IS**	WT-like	WT-like	WT-like	**PA2508**	6.0312799	n.d.
**PA2531::IS**	WT-like	WT-like	WT-like	**PA2531**	10.285887	n.d.
**PA3572::IS**	WT-like	WT-like	WT-like	**PA3572**	2.0353569	n.d.
**PA3914::IS**	WT-like	WT-like	WT-like	**PA3914**	12.007119	n.d.
**PA3573::IS**	WT-like	WT-like	WT-like	**PA3573**	3.5520207	2.00
**PA3581::IS**	WT-like	WT-like	WT-like	**PA3581**	2.0187101	n.d.
**PA3531::IS**	WT-like	WT-like	WT-like	**PA3531**	6.6984161	1.43
**PA4638::IS**	WT-like	WT-like	**Susceptible**	**PA4638**	2.4335741	n.d.
**PA5139::IS**	WT-like	WT-like	**Susceptible**	**PA5139**	7.033423	n.d.
**PA2171::IS**	WT-like	**Susceptible**	WT-like	**PA2171**	3.3116584	n.d.
**PA2313::IS**	WT-like	**Susceptible**	WT-like	**PA2313**	1.7541094	n.d.
**PA2184::IS**	WT-like (**hyper**)	**Susceptible**	**Susceptible**	**PA2184**	3.5862951	n.d.
**PA3915::IS**	Reduced	WT-like	**Susceptible**	**PA3915**	16.111408	n.d.
**PA0048::IS**	Reduced	WT-like	WT-like	**PA0048**	10.477965	2.84
**PA4913::IS**	Reduced	WT-like	WT-like	**PA4913**	3.1685878	3.83
**PA5033::IS**	Reduced	WT-like	WT-like	**PA5033**	5.2524326	n.d.
**PA0602::IS**	Reduced	**Susceptible**	WT-like	**PA0602**	5.7380729	n.d.
**PA0918::IS**	Reduced	**Susceptible**	WT-like	**PA0918**	4.3770897	2.62
**PA2114::IS**	Reduced	**Susceptible**	WT-like	**PA2114**	10.904505	4.55
**PA3236::IS**	Reduced	**Susceptible**	WT-like	**PA3236**	3.6714247	2.76
**PA4909::IS**	Reduced	**Susceptible**	WT-like	**PA4909**	8.7359344	n.d.
**PA5421::IS**	Reduced	**Susceptible**	WT-like	**PA5421**	4.4809046	1.57
**PA2146::IS**	Reduced	**Susceptible**	**Susceptible**	**PA2146**	19.654529	73.33

The relative transcript abundance in biofilms relative to planktonic cells for each of the genes is given, as determined by RNA-Seq and qRT-PCR. All experiments were carried out at least in duplicate.

Bold text highlights biofilm architecture or susceptibility phenotypes by mutant strains that differ from wild type.

^a^WT-like, appearance or susceptibility of biofilms formed by mutant strains was comparable to *P. aeruginosa* PAO1 biofilms.

^b^Reduced, apparent reduced biofilm biomass accumulation relative to *P. aeruginosa* PAO1 biofilms. The evaluation is based on visual inspection of the biofilm architecture, primarily taking into account the overall size/diameter of microcolonies relative to wild-type biofilms.

^c^Susceptible, the viability of biofilms formed by mutant strains was significantly reduced post-exposure to tobramycin or hydrogen peroxide relative to *P. aeruginosa* PAO1 biofilms.

^d^Biofilm susceptibility was determined using 150 µg/ml tobramycin or 0.6% hydrogen peroxide.

**Fig. 1:**
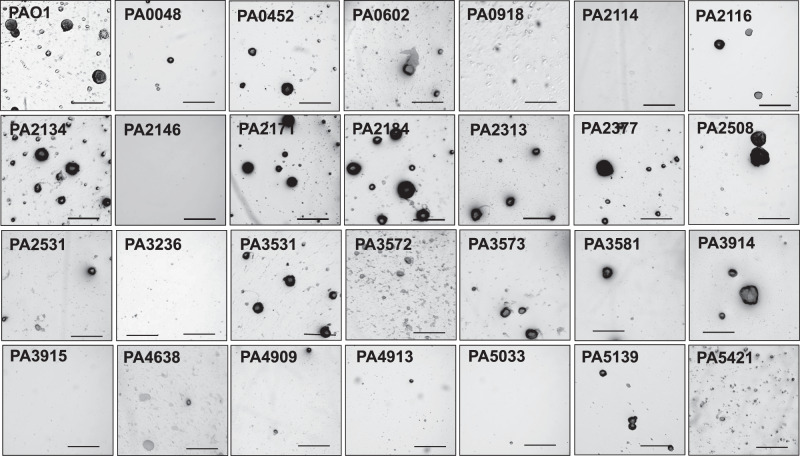
Contribution of genes encoding previously uncharacterized proteins to biofilm formation. Representative bright-field images of the biofilm architecture of wild-type and transposon mutants grown for 5 days in 24-well plates under semi-batch conditions. Size bar, 100 µm. All experiments were performed in triplicates.

To explore the contribution of genes encoding previously uncharacterized proteins in biofilm development, we selected 27 genes (hypothetical, uncharacterized, or not previously linked to biofilms) that were induced upon biofilm growth for further analysis (Table 1), by screening transposon mutants harboring insertional inactivation in these selected 27 genes for biofilm formation, with emphasis on their biofilm architecture. Biofilms were grown for 5 days in 24-well plates under semi-batch conditions, and the biofilm architecture subsequently evaluated by brightfield microscopy. *P. aeruginosa* PAO1 was used as the control. The visual analysis revealed a variety of biofilm structures, ranging from structured, wild-type like biofilms featuring large microcolonies to unstructured, flat surface-associated bacterial communities. Approximately half of the mutant strains formed biofilms similar in architecture to the wild-type PAO1, while the remaining mutant strains formed less structured or flat biofilms relative to the wild-type (Fig. 1). We furthermore evaluated the architecture of biofilms formed by a subset of the mutant strains under flowing conditions using confocal microscopy. We focused primarily on mutant strains that demonstrated less structured or flat biofilms relative to the wild type, to not only better visualize the biofilm architecture but also to ensure that differences in biofilm architecture were unrelated to the biofilm growth conditions (semi-batch versus flowing conditions). Overall, similar results were obtained for the architecture of biofilms formed by a subset of the mutant strains grown under flowing conditions using confocal microscopy (Supplementary Fig. 1). Our initial screen suggested 12 out of 27 selected genes to contribute to the formation of structured biofilms featuring microcolonies typically seen in *P. aeruginosa* biofilms, including genes PA0048, PA0602, PA0918, PA2114, PA2146, PA3236, PA3915, PA4909, PA4913, PA5033, and PA5421, while inactivation of PA2184 coincided with hyperbiofilm formation. A summary of the biofilm architecture formed by the mutant strains analyzed in this study is given in Table 1. It is of interest to note that differences in architecture are not due to differences in growth (Supplementary Fig. 2). Despite differences in the biofilm architecture, however, none of the mutant strains demonstrated impaired or reduced attachment behavior (Supplementary Fig. 3).

### Genes encoding previously uncharacterized proteins contribute to drug tolerance of *P. aeruginosa* biofilms

**Fig. 2:**
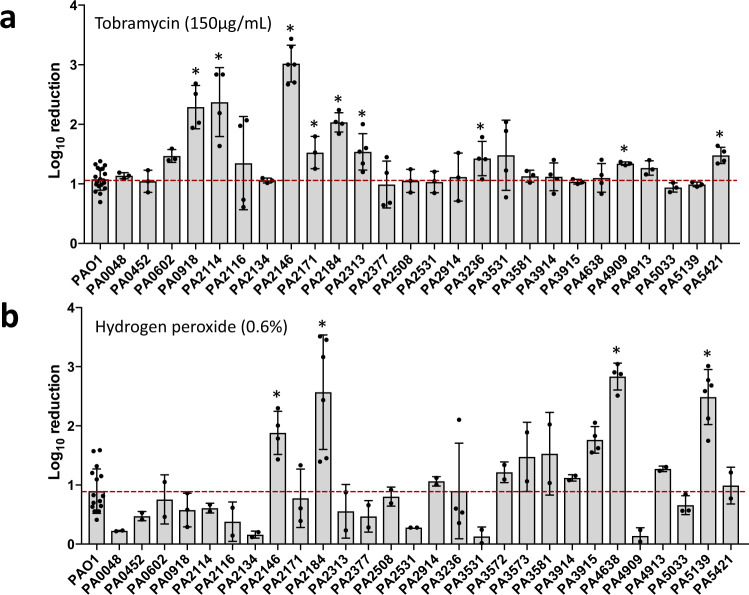
Contribution of genes encoding previously uncharacterized proteins to biofilm drug tolerance. Susceptibility phenotype of 2 day-old biofilms by wild type and transposon mutants to **a** tobramycin (150 µg/ml) and **b** 0.6% hydrogen peroxide. Biofilms were exposed to tobramycin or hydrogen peroxide for 1 h under flowing conditions. Viability was determined via CFU counts. Susceptibility is expressed as log_10_ reduction in viability. Experiments were performed in triplicates, with two technical replicates each. Error bars indicate standard deviation. Dashed lines indicate log reduction noted for wild-type biofilms. **, significantly different from PAO1; *p* < 0.1, as determined by ANOVA followed by a Dunnett’s post-hoc test.

We further surmised that genes demonstrating increased transcript abundance in biofilms may not only contribute to biofilm formation but may also play a role in *P. aeruginosa* biofilms gaining their heightened tolerance to antimicrobial agents or components of the immune system^3,27,28^. We therefore subjected biofilms formed by the same transposon mutants to tobramycin (150 ug/ml), a commonly used antibiotic in the treatment of *P. aeruginosa* infections^29,30^, or 0.6% hydrogen peroxide, to mimic a respiratory burst attack by macrophages and neutrophils^31^. Similar to the range of biofilm architectures, biofilms formed by mutant strains demonstrated a range of susceptibility relative to *P. aeruginosa* wild-type biofilms, ranging from less susceptible to more susceptible than wild-type biofilms (Fig. 2). Overall, our screen suggested that 13 out of 27 selected genes contribute to the susceptibility of *P. aeruginosa* biofilms to tobramycin and/or hydrogen peroxide. Mutant strains impaired in biofilm susceptibility to tobramycin only included those harboring insertional inactivations in PA0602, PA0918, PA2114, PA2171, PA2313, PA3236, PA3531, PA4909, and PA5421, while mutant strains impaired in biofilm susceptibility to hydrogen peroxide only included those harboring insertional inactivations in PA3915, PA4638, and PA5139. Mutant strains harboring insertional inactivations in PA2146 and PA2184 were impaired in biofilm susceptibility to both tobramycin and hydrogen peroxide. It is of interest to note that the susceptibility phenotype of these mutants was only apparent under biofilm growth conditions, as no difference in susceptibility was noted under planktonic growth conditions (Supplementary Fig. 4). A summary of the susceptibility phenotypes of mutant biofilms to tobramycin and hydrogen peroxide is given in Table 1.

### Factors affecting biofilm architecture and antibiotic susceptibility are predicted to form an interactome

**Fig. 3:**
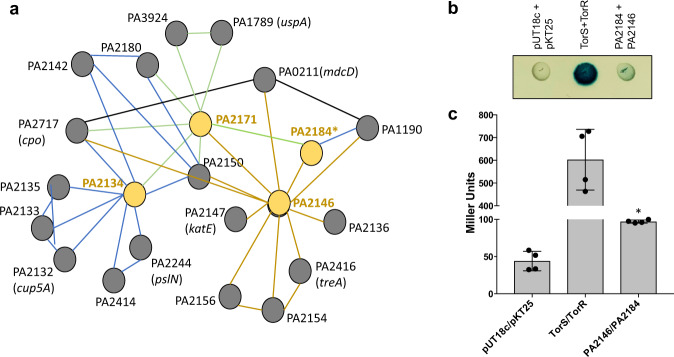
Predicted and confirmed protein-protein interactions by previously uncharacterized proteins that contribute to biofilm formation or biofilm drug tolerance. **a** Protein-protein interactions predicted by STRING (http://string-db.org). Proteins found to affect biofilm formation and/or susceptibility are shown in yellow. Green lines indicate interactions found for PA2171, brown lines for PA2146, and blue lines for PA2134. *, PA2184 is absent from the database in STRING version 11.0. Proteins shown to interact by STRING are predicted to be located in the cytoplasm, based on pseudomonas.com. **b** Detection of a physical interaction between PA2146 and PA2184 by bacterial two-hybrid analysis. The PA2146 gene was cloned into the vector pKT25, and PA2184 into the vector pUT18C. Plasmids were co-transformed into *E. coli* DHM1 cells. The transformants were spotted (2 µl) onto LB agar containing ampicillin, kanamycin, IPTG, and X-Gal. *E. coli* harboring empty vectors was used as negative control while cells producing TorR and TorS were used as positive control. Plates were incubated at 30°C for 48 h. Cleavage of X-Gal (blue) indicates a positive protein-protein interaction. Representative image is shown. Additional images and controls are shown in Supplementary Fig. 5a. **c** Quantitative analysis of protein-protein interactions, by *E. coli* DHM1 harboring pUT18C + pKT25, TorR+TorS, and PA2146 + PA2184, as determined using the Miller assay. Experiments were carried out in triplicates but only representative image is shown. Additional images and controls are shown in Supplementary Fig. 5a. Error bars indicate standard deviation. **significantly different from *E. coli* DHM1 harboring empty vectors (pUT18C + pKT25, *p* < 0.05, as determined by ANOVA followed by a Dunnett’s post-hoc test.

Our screen revealed 12 out of 27 genes to contribute to the formation of structured biofilms, while 13 out of 27 genes contributed to the susceptibility phenotype of *P. aeruginosa* biofilms, with 8 genes affecting both biofilm formation and antibiotic susceptibility (Table 1). The findings suggested biofilm formation and antibiotic susceptibility to be somewhat linked. To elucidate possible functional partnerships, we made use of STRING (http://string-db.org), a biological database and web resource of known and predicted protein–protein interactions. Several interactions based on gene neighborhoods were predicted to include interactions between PA2146 and PA2171, components of probable ABC transporters PA4909 and PA4913^32^, PA3915, and PA3914 as well as PA3581 and PA3582. Additionally, we surveyed the predicted interactomes of each of the proteins of interest and found PA2134, PA2146, PA2171, and PA2184 to be predicted to form extensive and overlapping interactomes (Fig. 3a). In addition to PA2171 and PA2184, the predicted interactome of PA2146 included proteins involved in metabolic processes (TreA and MdcD), as well as enzymes harboring catalase or peroxidase activity (Cpo/PA2717, KatE) (Fig. 3a). Predicted PA2171 interaction partners included proteins linked to redox reactions, stress, and repair of DNA double-strand breaks by non-homologous end-joining (PA2140 and PA2180, PA1789, PA2150, respectively). In addition, PA2171 was predicted to directly interact with PA2146, PA2134, and PA2184. The PA2134 interactome comprised proteins encoding chaperone CupA5, as well as proteins involved in transport (PA2135) and metabolic processes (PA2171, PA2142, PA2414).

To confirm STRING predicted interactions, we made use of a bacterial adenylate cyclase‐based two‐hybrid (BACTH) assay of *E. coli* to probe for interactions between PA2146 and PA2184, two previously uncharacterized proteins that primarily contribute to biofilm drug tolerance (Fig. 2, Table 1). Full-length proteins were fused to either the T18 or the T25 subunit of adenylate cyclase and then co-expressed in *E. coli* DHM1 to test for interaction. For controls, we made use of TorR and TorS^33^, as well as a control strain harboring empty vectors (pKT25, pUT18c). A positive interaction between proteins was detected as blue colonies on medium containing X-Gal (5-bromo-4-chloro-3-indolyl-β-D-galactopyranoside); deeper blue indicates a stronger interaction. We observed a positive interaction between the positive control proteins TorR and TorS, while no color change was noted for the vector control strain (Fig. 3b, Supplementary Fig. 5a). Moreover, a positive interaction was noted between PA2146 and PA2184. The interaction was confirmed using Miller assays (Fig. 3c, Supplementary Fig. 5a). In addition, an interaction between PA2146 and PA2184 was detected using pulldown assays (Supplementary Fig. 5b).

### Factors affecting biofilm architecture and antibiotic susceptibility

Given the potential interaction of proteins contributing to biofilm formation and/or antibiotic susceptibility, we furthermore explored whether the respective 16 genes have been reported to be differentially expressed upon transition to the surface associated mode of growth by *P. aeruginosa* PAO1 and clinical isolates when grown in varying growth medium, and in vitro and in vivo biofilm models. Doetsch et al.^34^ evaluated the *P. aeruginosa* PA14 transcriptome in planktonic cultures and biofilms grown in 96-well plates in LB for 48 h under static conditions. The study revealed PA2114, PA2146, PA2171, PA3236, PA4909 and PA5421 to be significantly increased in static biofilms relative to planktonic cells grown to stationary phase, while PA3531 was found to be significantly decreased relative to planktonic growth conditions (Supplementary Table 3). With the exception of PA2184, Thöming et al.^35^ identified the genes of interest as part of the core genome obtained by comparing the genome of 77 clinical isolates (Supplementary Table 3). Moreover, the study revealed that 13 of the 16 genes increased in at least one of the three clusters of major biofilm phenotype formers, with PA2146 being significantly increased upon biofilm formation in all 77 clinical isolates (Supplementary Table 3)^35^. The study by Turner et al.^36^ aimed at investigating the genetic requirements for acute and chronic pathogenesis in *P. aeruginosa* infections, by identifying genes that are increased in biofilms present in burn and chronic surgical wound infection relative to biofilms grown in succinate-Mops minimal medium. The transcriptome analysis revealed all 19 genes identified in this study were found to be increased in burn and chronic surgical wound infection, with 11 out of 19 genes being significantly increased in in vivo relative to in vitro biofilms (Supplementary Table 3), with genes PA2146, PA2171 and PA2184 demonstrating the largest increase. Bielecki et al.^37^ likewise noted these three genes to be increased in burn wound infection, further noting that genes within the region of PA2134 – PA2190 may possibly contribute to the accumulation and breakdown of storage materials such as glycogen and trehalose (*glgA, glgB, glgP*), in protection against oxidative stress like the catalase encoding gene *katE*, or in general stress response. In comparison, only a subset of the 16 genes were found to be induced by *P. aeruginosa* when grown in soft tissue and CF sputum (Supplementary Table 3)^38^. Overall, genes identified in this study that were most frequently found to be induced under biofilm growth conditions, both in vitro and in vivo, included PA2146, PA2171, PA2313, PA3236, PA3531, PA3573, PA3915, PA4909, PA4913, PA5033, PA5139, and PA5421. Among these, PA2146 was the most consistently and most significantly upregulated genes (Supplementary Table 3).

### PA2146 encodes a small protein that is prevalent in various Gram-negative and Gram-positive bacterial species and highly expressed in biofilms formed by *P. aeruginosa* laboratory and clinical isolates

**Fig. 4:**
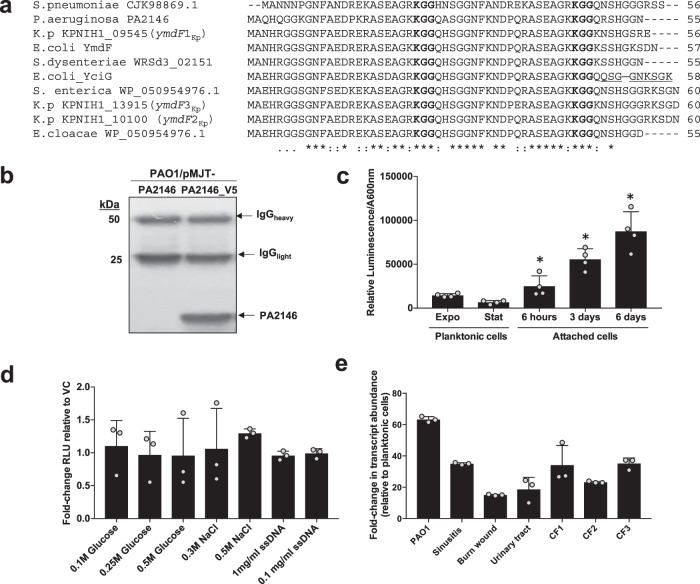
PA2146 encodes a conserved, small protein that is expressed under surface-associated growth conditions in *P. aeruginosa* PAO1 and clinical isolates. **a** Sequence alignment of the *P. aeruginosa* PA2146 and homologs from various ɣ-proteobacteria and the gram-positive *Streptococcus pneumoniae*. “*“ denotes identical amino acids. For comparison, the *E. coli* YciG protein sequence harboring the KGG motif followed by the Walker nucleotide binding motif (highlighted in bold) is shown below the alignment in blue. **b** Detection of V5-tagged PA2146 in *P. aeruginosa* PAO1 via immunoprecipitation and immunoblotting using anti-V5 antibodies. PAO1 producing untagged PA2146 was used as a negative control. IgG_heavy/light_ refer to antibody bands. Experiments were carried out in triplicates but only representative image is shown. The image of the uncropped blot is shown in the supplementary material. **c**–**e** Assessment of PA2146 promoter reporter activity as determined by measuring luminescence. The putative promoter region of PA2146 (−513 to −1 relative to the translational start site) was cloned into the mini-CTX-*lux* vector to create CTX-PA2146-*lux*, which was introduced into the wild-type *P. aeruginosa* PAO1 strain. A promoterless *lux* strain was used as control. Background levels of luciferase activity observed for the control PAO1 bearing the promoterless *lux* operon were subtracted from the corresponding readings for PAO1::*P*_*PA2146*_*513-lux* and normalized to total cells (A600 nm). All experiments were carried out in triplicate. Error bars indicate standard deviation. **c** Luminescence of the resulting strains was measured in planktonic cells grown to exponential (Expo) and stationary phase (Stat), and cells attached to the surface for 6 h (6 h), 3 days (3d), or 6 days (6d). *significantly different from exponential stage planktonic cells, *p* < 0.05, as determined by ANOVA followed by a Dunnett’s post-hoc test. **d** Relative fluorescence units of the reporter strain grown planktonically in the absence and presence of sperm DNA (0.1–1 mg/ml), 0.1–0.5 M glucose, and 0.3–0.5 M sodium chloride. No significant different was noted based on ANOVA followed by a Dunnett’s post-hoc test. **e** Fold-increase in transcript abundance of PA2146 in biofilms formed by *P. aeruginosa* PAO1 and clinical isolates relative to the same strains grown planktonically. All strains were grown planktonically to mid-log stage and as biofilms for 3 days. Experiments were performed using biological triplicates. Error bars indicate standard deviation.

Of the genes that were most frequently found to be induced under biofilm growth conditions by us and others^34–38^, genes encoding PA2146 and PA2171 (and PA2184) are predicted to likely interact and work in concert (Fig. 3) and to contribute to biofilm formation and/or biofilm drug tolerance. However, little is known about these proteins, with the Pseudomonas genome database annotating PA2146, PA2171, and PA2184 as (conserved) hypothetical proteins^32^. However, homologs of PA2146 have been described in various Gram-negative and Gram-positive species. For example, a PA2146 homolog contributes to motility and stress response in *Salmonella enterica*^39,40^, with the expression being dependent on RpoS^40^. In *Escherichia coli*, a PA2146 homolog has been reported to be inducible by glucose starvation and other stressors^41^. An alignment of select PA2146 homologs sharing more than 80% identity and harboring a KGG repeat motif typically found in proteins that are expressed under conditions of stress in bacteria, is shown in Fig. 4a. Phylogenetic tree analysis furthermore suggested that PA2146 is evolutionarily derived from an ancestor within the *Enterobacteriaceae*. It forms part of a clade of sequences from *Pseudomonas* taxa that nests with very high probability within a group of diverse *Enterobacteriaceae* genera (*Escherichia, Enterobacter, Citrobacter, Cedecea, Leclercia, Lelliottia, Shigella*), with PA2146 sharing common ancestry with the *Enterobacteriaceae* homologs in *K. pneumoniae* and *E. coli* (Supplementary Fig. 6).

In *P. aeruginosa*, PA2146 is predicted to encode a small cytoplasmic protein of 55 amino acid (5.8-kDa)^32^. Using a C-terminally V5-tagged PA2146 construct, we confirmed that PA2146 is produced as in *P. aeruginosa* (Fig. 4b). Given the lack of a Walker nucleotide binding motif, we next made use of a luciferase-based transcriptional reporter system to elucidate the expression pattern of PA2146 in *P. aeruginosa*. Luciferase activity started increasing in *P. aeruginosa* cells as early as 6 h following initial attachment to the surface relative to the activity observed in exponential phase cells, and continued increasing with prolonged biofilm growth through day 6 (Fig. 4c). As biofilm growth has been reported to be similar to growth in stationary phase, we also compared PA2146 promoter activity in the different stages of planktonic growth. Interestingly, however, luciferase activity decreased in stationary phase PAO1::*P*_*PA2146*_*513-lux* cells relative to exponential phase cells (Fig. 4c), suggesting that slowed growth or stressful conditions prevalent in stationary phase are likely not responsible for PA2146 up-regulation upon transition to biofilm growth. We furthermore explored whether presence of eDNA and increased salt and glucose concentrations coincided with induction of PA2146 under planktonic conditions. eDNA was chosen due to its prevalence in biofilms as well as its chelating properties^42,43^, while we chose salt and glucose to induce general stress. However, neither the presence of eDNA, glucose or sodium chloride coincided with increased PA2146 promoter activity relative to the vector control strain (PAO1::Lux) (Fig. 4d), further suggesting that in *P. aeruginosa*, PA2146 expression is primarily linked to surface associated growth conditions.

Given that PA2146 appeared to be induced upon biofilm growth, and not by stressful conditions, we next explored whether this induction was limited to biofilms formed by the laboratory PAO1 strain or linked to biofilm formation in general, using qRT-PCR. We therefore determined PA2146 transcript abundance in biofilms formed by *P. aeruginosa* PAO1 and six *P. aeruginosa* clinical strains previously isolated from various infection sites, including sinuses, the urinary tract, burn wounds, and the lungs of cystic fibrosis patients. Similarly to PAO1, the clinical isolates exhibited 10- to 50-fold elevated PA2146 transcript abundance under biofilm growth conditions relative to planktonic cells (Fig. 4e), suggesting a biofilm-specific expression pattern of PA2146 in *P. aeruginosa*. Our results are in agreement with findings by Thöming et al.^35^.

### PA2146-like genes affect biofilm formation and antibiotic susceptibility in *E. coli* and *K. pneumoniae*

As homologs of PA2146 are not limited to *P. aeruginosa* but are conserved in diverse bacterial species including ɣ-proteobacteria (Fig. 4a, Supplementary Fig. 6a), we next asked whether PA2146 homologs likewise contribute to biofilm formation and biofilm drug tolerance in bacterial species other than *P. aeruginosa*. We therefore chose *E. coli* BW25113 and *K. pneumoniae* KPNIH1. The two biofilm-forming organisms were chosen due to their marked differences in biofilm architecture, biofilm matrix polysaccharides, and cell-to-cell signaling systems^44–50^. Moreover, *K. pneumoniae* is considered an ESKAPE pathogen responsible for hospital acquired pneumonia and urinary tract infections^51^, while *E. coli* is responsible for many common bacterial infections, including bacteremia and urinary tract infections^52–54^.

**Fig. 5:**
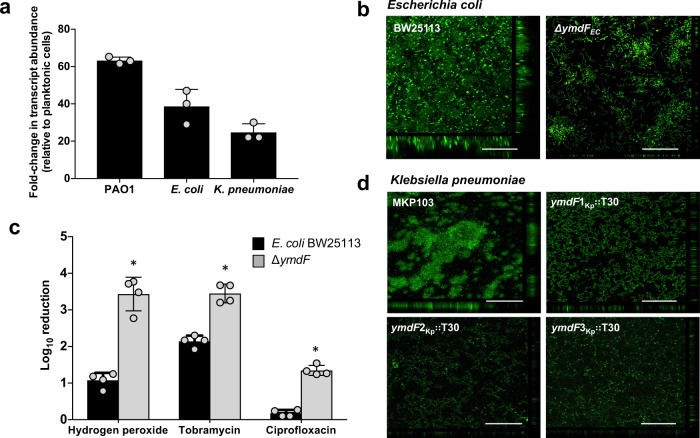
PA2146 homologs contribute to biofilm formation and antibiotic susceptibility of biofilms formed by *K. pneumoniae* and *E. coli*. **a** Fold-increase in transcript abundance of PA2146 and PA2146 homologs in biofilms formed by *P. aeruginosa* PAO1, *E. coli* BW25113, and *K. pneumoniae*, relative to the same strains grown planktonically. All strains were grown planktonically to mid-log stage. Biofilms formed by *P. aeruginosa* were grown for 3 days, while biofilms by *E. coli* and *K. pneumoniae* were grown for 4 days. Experiments were performed using biological triplicates. Error bars indicate standard deviations. **b** Representative confocal images of biofilms formed by *E. coli* wild type and isogenic mutant strain ∆*ymdF* grown for 5 days under continuous flowing conditions in flow cells. Biofilm architecture was visualized via confocal microscopy following *Bac*Light LIVE/DEAD staining. White bar, 100 µm. **c** Antimicrobial susceptibility of 3-day old biofilms formed by *E. coli* wild type and the isogenic mutant strain ∆*ymdF* to hydrogen peroxide (0.3%), tobramycin (75 μg/ml), and ciprofloxacin (150 μg/ml). Biofilms were exposed to the antimicrobial agents for 1 h under flowing conditions. Viability was determined via CFU counts. Susceptibility is expressed as log_10_ reduction in CFU counts. Experiments were performed using biological triplicates. Error bars indicate standard deviations. *significantly different from *E. coli* BW25113, *p* < 0.05, as determined by ANOVA followed by a Dunnett’s post-hoc test. **d** Representative confocal images of biofilms formed by *K. pneumoniae* wild type and isogenic mutant strains *ymdF*1_Kp_∷T30, *ymdF*1_Kp_∷T30, and *ymdF*1_Kp_∷T30 grown for 5 days under semi-batch conditions in microtiter plates. Biofilm architecture was visualized via confocal microscopy following *Bac*Light LIVE/DEAD staining. White bar, 100 µm.

Interestingly, most *K. pneumoniae* strains contain either two or three homologs of PA2146 (inparalog), with *K. pneumoniae* KPNIH1 encoding three (here referred to as *ymdF*1_Kp_, *ymdF*2_Kp_, and *ymdF*3_Kp,_ (Fig. 4a, Supplementary Fig. 6a,b). Likewise, *E. coli* BW25113 harbors two PA2146 homologs, namely *ymdF* and *yciG* (Fig. 4a, Supplementary Fig. 6a,b). YciG differs from YmdF, however, by the presence of a Walker nucleotide binding motif (underlined sequence, QSGGNKSGKS, in Fig. 4a). Likewise, *P. aeruginosa* PAO1 harbors an inparalog of PA2146, namely PA2190, which is approximately two times longer than the PA2146 genes, likely due to a duplication event (Fig. 4a, Supplementary Fig. 6c). However, this inparalog was not further analyzed due to the apparent difference in length. Using qRT-PCR, we confirmed increased transcript abundance of PA2146 homologs in biofilms formed by *E. coli* and *K. pneumoniae* relative to cells grown planktonically (Fig. 5a). Interestingly, the trend was not exclusive to PA2146 and its homologs: transcript abundance of homologs of *P. aeruginosa* genes found to up-regulated upon biofilm growth were likewise increased in a biofilm-specific manner in *E. coli* (Table 2).

**Table 2:** Relative transcript abundance of *P. aeruginosa* genes and the respective *E. coli* homologs in biofilms relative to mid-exponential stage planktonic cells.

GENE		Relative expression^a^		
*P. aeruginosa*	*E. coli*	*P. aeruginosa*		*E. coli*
		RNA-seq^b^	qRT-PCR	qRT-PCR
PA2146	*ymdF*	19.70	73.33 (±11.63)	38.90 (±9.99)
PA3531; *bfrB*	*Bfr*	9.65	5.64 (±3.30)	3.36 (±0.33)
PA5355; *glcD*	*glcD*	4.89	3.00 (±1.41)	1.74 (±0.25)
PA3584; *glpD*	*glpD*	7.88	15.56 (±10.45)	16.99 (±1.77)
PA3581; *glpF*	*glpF*	2.01	8.49 (±1.46)	12.72 (±3.11)
PA2184	*yciE*	3.58	6.19 (±0.56)	91.89 (±17.63)
PA0529	*yiiM*	2.46	10.40 (±3.24)	1.94 (±0.05)
PA0905; *rsmA*	*csrA*	2.38	3.58 (±0.67)	1.96 (±0.20)

^a^Fold-change in relative abundance of *P. aeruginosa* transcripts as determined via RNA-seq or qRT-PCR, and of *E. coli* transcript as determined via qRT-PCR. Standard deviation for qRT-PCR is listed in parentheses. *N.D.*, not determined.

^b^All included RNA-seq fold-changes have been determined to be significant by edgeR (*p* < 0.05).

We next elucidated the role of PA2146 homologs in biofilm formation. Flow cell-grown wild-type *E. coli* BW25113 biofilms were characterized by a substantial biofilm thickness, a confluent substratum coverage, and the presence of cellular aggregates. In contrast, a mutant strain inactivated in the PA2146 homolog *ymdF*_,_ failed to develop the typical three-dimensional biofilm architecture, only forming a patchy monolayer (Fig. 5b, Table 3). The difference in biofilm biomass accumulation was confirmed by viability counts, with biofilms formed by the wild-type *E. coli* strain harboring 5.0 × 10^8^ ± 4.2 × 10^8^ CFU/mL, whereas *ΔymdF* biofilms comprised 10-times fewer cells (~5.5 × 10^7^ ± 5.3 × 10^7^ CFU/mL). We did not evaluate the role of YciG due to the presence of the Walker nucleotide-binding motif. Inactivation of either of the three PA2146 inparalogs in *K. pneumoniae* likewise negatively affected *K. pneumoniae* biofilm formation, with all three mutant strains demonstrating impaired architecture development and close to 100-fold reduced biomass accumulation, as evidenced by, microscopy, COMSTAT analysis, and CFU determinations (Fig. 5d, Table 3).

**Table 3:** COMSTAT 2.0 quantitative analysis of CSLM images of biofilms formed by indicated strains.

	Biomass (µm^3^/µm^2^)	Thickness (µm)
*P. aeruginosa*		
PAO1	6.52 (±3.26)	5.82 (±5.31)
PAO1/pMJT-1	7.28 (±3.1)	8.25 (±13.12)
PAO1/pMJT- PA2146	13.84 (±7.16)*	15.61 (±26.32)*
ΔPA2146	1.09 (±0.97)	1.4 (±1.61)
ΔPA2146/pMJT-1	0.46 (±0.18)*	0.51 (±1.3)*
ΔPA2146/pMJT-PA2146	5.43 (±3.29)	4.76 (±7.63)
ΔPA2146/pMJT-*ymdF*_*Ec*_	9.02 (±6.4)	11.41 (±9.36)
ΔPA2146/pMJT-*ymdF*1_Kp_	10.35 (±2.72)	15.32 (±10.50)
ΔPA2146/pMJT-*ymdF*2_Kp_	11.01 (±3.57)	13.94 (±5.37)
ΔPA2146/pMJT-*ymdF*3_Kp_	8.30 (±3.77)	9.82 (±4.63)
ΔPA2146/pMJT-*sagS*	1.76 (±2.3)*	1.95 (±8.29)*
Δ*sagS*	0.82 (±0.63)*	0.83 (±2.88)*
Δ*sagS*/pMJT-1	0.62 (±0.5)*	0.52 (±3.61)*
Δ*sagS*/pMJT-PA2146	9.24 (±6.4)	8.96 (±23.64)
Δ*sagS*/pMJT-*ymdF*	7.76 (±5.44)	11.99 (±23.01)
Δ*sagS*/pMJT-*ymdF*1_Kp_	8.18 (±3.34)	9.52 (±4.22)
Δ*sagS*/pMJT-*ymdF*2_Kp_	8.28 (±2.39)	9.27 (±2.85)
Δ*sasgS*/pMJT-*ymdF*3_Kp_	9.67 (±3.19)*	11.27 (±4.48)*
*E. coli*		
BW25113	7.22 (±1.51)	8.70 (±1.76)
Δ*ymdF*_Ec_	1.19 (±0.75)*	0.94 (±0.64)*
*K. pneumoniae*		
MKP103	5.36 (±1.85)	6.57 (±3.29)
*ymdF*1_Kp_∷T30	1.76 (±0.77)*	3.97 (±2.33)*
*ymdF*2_Kp_∷T30	0.60 (±0.49)*	1.07 (±1.09)*
*ymdF*3_Kp_∷T30	0.31 (±0.32)*	0.81 (±1.65)*

Experiments were performed in triplicate, with each biological replicate being represented by a minimum of 8 confocal images taken at random. Standard deviation is indicated in parenthesis. *Significantly different from respective parental background (PAO1, PAO1/pMJT-1, BW25113, or MKP103); *p* < 0.05, as determined using 1-way ANOVA, followed by a Dunnett’s post-hoc test.

To determine whether inactivation of PA2146 homologs likewise coincides with increased susceptibility to antimicrobial agents, we next assessed the susceptibility of *E. coli* and *K. pneumoniae* biofilms to antimicrobial agents. Biofilms formed by *E. coli* were exposed to hydrogen peroxide, tobramycin, and ciprofloxacin. Exposure of *E. coli* wild-type biofilms to hydrogen peroxide for 1 h coincided with a 1-log reduction in viable biofilm cells, whereas the same treatment reduced the viability of *∆ymdF* biofilms by 2.9 logs, suggesting a 100-fold difference in susceptibility (Fig. 5c). Exposure to tobramycin and ciprofloxacin likewise coincided with a significant reduction in the viability of *∆ymdF* biofilms relative to wild-type biofilms (Fig. 5c). Similarly, biofilms formed by *K. pneumoniae* wild-type and mutants were exposed to gentamicin or colistin, two antibiotics that are commonly used against multi-resistant carbapenemase-producing *K. pneumoniae*. While the viability of mutant biofilms was consistently and significantly reduced following antibiotic treatment relative to wild-type *K. pneumoniae* biofilms (data not shown), the overall low biofilm biomass of the mutant strains relative to wild-type biofilms prevented accurate and meaningful comparisons via susceptibility assays.

### *E. coli* and *K. pneumoniae* PA2146 homologs can substitute for PA2146 in *P. aeruginosa*

**Fig. 6:**
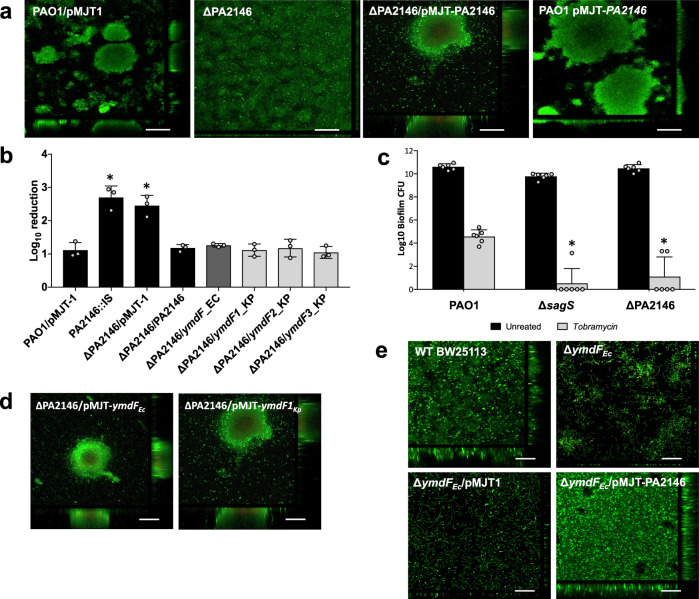
Expression of PA2146 and PA2146 homologs restores the phenotypes of biofilms formed by *P. aeruginosa* ΔPA2146 *and E. coli ΔymdF* to wild-type levels. **a** Representative confocal images of biofilms formed by PAO1/pMJT-1, ∆PA2146, ∆PA2146/pMJT-PA2146, and PAO1/pMJT-PA2146 grown for 5 days under continuous flowing conditions in flow cells. Prior to confocal imaging, biofilms were stained with *Bac*Light LIVE/DEAD-stain. Size bar, 100 µm. **b** Susceptibility of biofilms formed by *P. aeruginosa* PAO1 and ∆PA2146 mutant strains harboring an empty vector (pMJT-1) or overexpressing PA2146 or homologs of PA2146 from *E. coli* (*ymdF*_EC) and *K. pneumoniae* (*ymdF1*_KP, *ymdF2*_KP, *ymdF3*_KP). Viability was determined via CFU counts. Killing is shown as log_10_ reduction. Experiments were performed at least in triplicates, with each repeat comprising two technical replicates. *, significantly different from tobramycin-treated PAO1 biofilms, *p* < 0.05, as determined by ANOVA followed by a Dunnett’s post-hoc test. **c** Antimicrobial tolerance of biofilms formed by PAO1, ∆*sagS* and ∆PA2146, as determined using biofilm-MBC assays. Biofilms were grown for 3 days continuous flowing conditions in tube reactors, followed by exposure to medium alone (untreated) or tobramycin (100 μg/ml) for 24 h under flowing conditions. Viability was determined via CFU counts. Experiments were performed at least in triplicates, with each repeat comprising four technical replicates. **, significantly different from tobramycin-treated PAO1 biofilms, *p* < 0.001, as determined by ANOVA followed by a Dunnett’s post-hoc test. **d** Representative confocal images of biofilms formed by ∆PA2146 overexpressing PA2146 homologs from *E. coli* (*ymdF*_Ec_) and *K. pneumoniae* (*ymdF1*_Kp_). Biofilms were grown for 5 days under continuous flowing conditions in flow cells. Prior to confocal imaging, biofilms were stained with *Bac*Light LIVE/DEAD-stain. **e** Representative confocal images of biofilms formed by *E. coli* wild type and isogenic mutant strain ∆*ymdF*, as well as ∆*ymdF* harboring the empty vector pMJT-1 or overexpressing PA2146. Biofilms were grown for 5 days under continuous flowing conditions in flow cells. Biofilm architecture was visualized via confocal microscopy following *Bac*Light LIVE/DEAD staining.

Based on the roles of the PA2146 homologs in *E. coli* and *K. pneumoniae*, we reasoned that PA2146 homologs from these strains can substitute for PA2146 in *P. aeruginosa*, apparent by restoration of biofilm phenotypes to wild-type levels. To address these questions, we first generated a clean deletion mutant of ∆PA2146. Similar to the transposon mutant PA2146::IS (Fig. 1), the isogenic ∆PA2146 mutant formed biofilms that lacked large cellular aggregates and demonstrated 2-fold reduced biofilm biomass and thickness relative to the wild-type biofilms that were characterized by a three-dimensional architecture dominated by large microcolonies (Fig. 6a, Table 3). Multicopy expression of PA2146 restored biofilm formation by ∆PA2146 to wild-type levels (Fig. 6a, Table 3). Moreover, biofilms formed by ∆PA2146 demonstrated increased susceptibility to tobramycin relative to the wild type following 1 h of exposure (Fig. 6b). Considering the increased susceptibility of ∆PA2146 biofilms to tobramycin, we also asked if PA2146 contributes to the tolerance or recalcitrance of biofilm cells to killing by antimicrobial agents^55,56^. To do so, we subjected 3-day-old biofilms to biofilm-MBC assays, exposing biofilm cells to 100 µg/ml tobramycin for 24 h under flowing conditions. Relative to wild-type biofilms, ∆PA2146 biofilms demonstrated a significantly reduced tolerance to tobramycin post 24 h (Fig. 6c). A similar reduction in tolerance was noted for ∆*sagS* biofilms which were previously confirmed to be impaired in the tolerance to tobramycin^57,58^. It is important to note that PA2146 inactivation did not affect *P. aeruginosa* growth rate or minimum inhibitory concentrations (MIC) of planktonic cells (Supplementary Fig. 7).

Multi-copy expression of *E. coli ymdF* in *P. aeruginosa* ΔPA2146 rescued the biofilm architecture and biofilm susceptibility to tobramycin to wild-type levels (Fig. 6b, d, Table 3). Likewise, multi-copy expression of any of the *K. pneumoniae ymdF* inparalogs in *P. aeruginosa* ΔPA2146 restored the ΔPA2146 biofilm architecture to wild-type levels (Fig. 6b, d, Table 3). Moreover, restoration of biofilm architecture was not limited to *P. aeruginosa*, as multicopy expression of PA2146 also rescued the architecture of *E. coli ΔymdF* biofilms to wild-type levels (Fig. 6e, Table 3).

### *E. coli* CsrA does not restore biofilm formation by a *P. aeruginosa ∆rsmA* mutant to wild-type levels

**Fig. 7:**
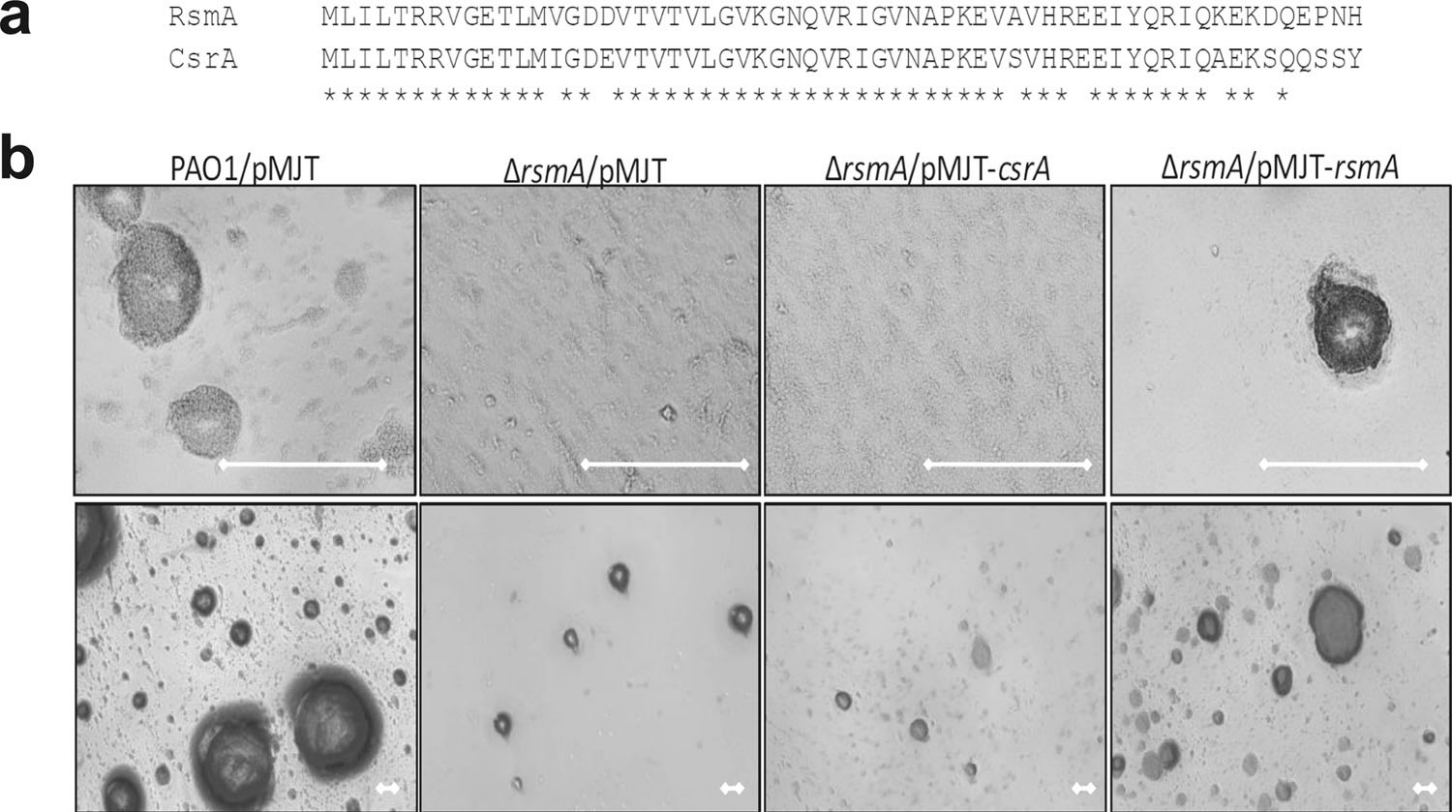
*E. coli* CsrA does not cross-complement for *P. aeruginosa* RsmA. **a** Alignment of RsmA and CsrA protein sequences. **b** Biofilm formation of indicated strains grown for 6 days in 24-well plates was assessed using brightfield microscopy. Experiments were performed using biological triplicates, with representative images shown. White bar, 100 μm.

Considering the sequence and functional conservation between *P. aeruginosa* PA2146, the *E. coli* YmdF and the YmdF inparalogs in *K. pneumoniae*, we next asked whether proteins that are highly conserved in sequence are interchangeable in general. To address this question, we made use of *P. aeruginosa* posttranscriptional regulator RsmA and its *E.coli* counterpart, the posttranscriptional regulator CsrA^59–62^. RsmA belongs to the CsrA/RsmA family of proteins that act by modulating translation initiation at target mRNAs using post‐transcriptional regulatory mechanisms^60,62,63^. Considering that both the *P. aeruginosa* RsmA and the *E. coli* CsrA play similar roles in biofilm formation and are highly similar in sequence (85% identity, Fig. 7a)^59–62,64^, we analyzed the biofilm architecture of cross-complemented strains. An *∆rsmA* mutant, demonstrating reduced biofilm formation, was rescued by expression of *rsmA*, but not of *csrA* (Fig. 7b)*. ∆rsmA* has an aggregative phenotype when grown planktonically in liquid. Similar to biofilm formation, expression of *rsmA* restored the *∆rsmA* hyperaggregative phenotype to wild-type levels, while *∆rsmA* expressing *csrA* remained hyperaggregative (not shown). The findings suggested that RsmA and CsrA are not interchangeable. Lack of restoration is likely due to RsmA and CsrA targeting different EPS-associated mRNAs and by being antagonized by dissimilar small RNAs^59,65–67^.

### PA2146 likely functions downstream of SagS and contributes to biofilm c-di-GMP levels and BrlR abundance

**Fig. 8:**
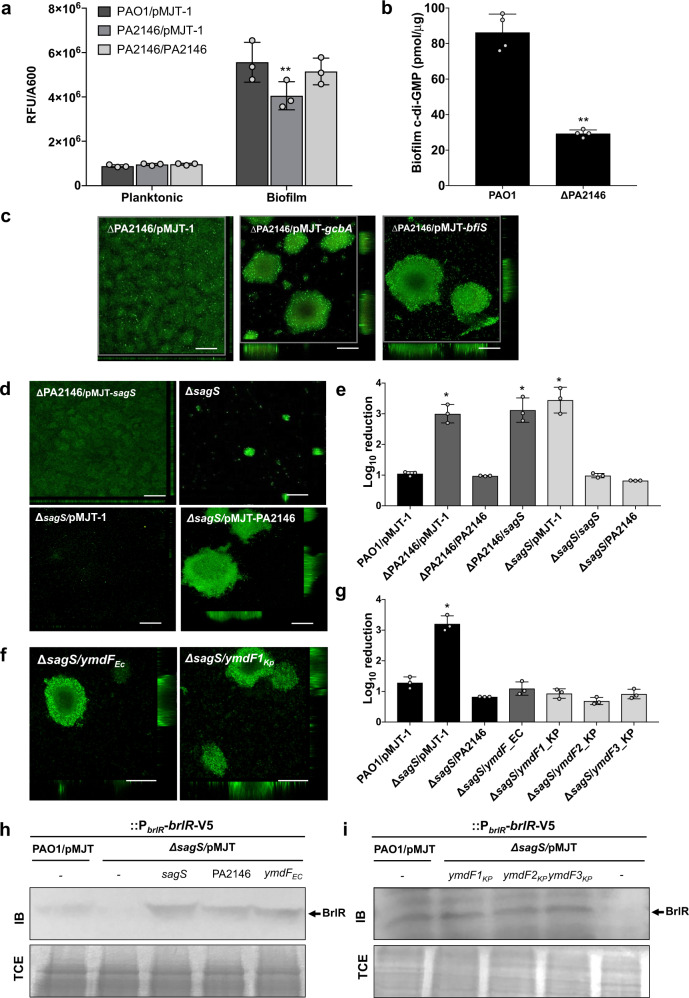
Multi-copy expression of PA2146 and PA2146 homologs by *K. pneumoniae* and *E. coli* rescue the *P. aeruginosa ΔsagS* biofilm phenotypes to wild-type levels. **a** Relative levels of intracellular c-di-GMP present planktonic and biofilm cells formed by wild-type *P. aeruginosa* PAO1, ∆PA2146, and ∆PA2146 expressing PA2146 and harboring the unstable c-di-GMP reporter pCdrA::*gfp*(ASV). Planktonic cells were grown in LB to exponential phase while biofilms were grown in 24-well plates for 3 days. The relative fluorescence (RFU) was normalized to A600nm. Experiments were performed using biological triplicates. Error bars indicate standard deviations. *significantly different from PAO1/pMJT-1, *p* < 0.05, as determined by ANOVA followed by a Dunnett’s post-hoc test. **b** c-di-GMP levels present in wild-type and ∆PA2146 mutant biofilm cells. The strains were grown in tube reactors under flowing conditions for 5 days prior to c-di-GMP extraction and quantitation by HPLC analysis. pmol/mg refers to c-di-GMP levels (pmol) per total cell protein (in mg). Experiments were performed using biological triplicates. Error bars indicate standard deviations. *, significantly different from PAO1, *p* < 0.05, as determined using Student’s *t*-test. **c** Representative confocal images of biofilms formed by ∆PA2146/pMJT-1, ∆PA2146/pMJT-*gcbA*, and ∆PA2146/pMJT-*bfiS* grown for 5 days under continuous flowing conditions. Prior to confocal imaging, biofilms were stained with *Bac*Light LIVE/DEAD-stain. Size bar, 100 µm. **d** Representative confocal images of biofilms formed by PAO1, ∆sagS and ∆PA2146 harboring an empty vector or expressing PA2146 or *sagS*. Biofilms were grown for 5 days under flowing conditions. Size bar, 100 µm. **e** Antimicrobial susceptibility of biofilms formed by PAO1, ∆*sagS* and ∆PA2146 harboring an empty vector or expressing PA2146 or *sagS* to tobramycin. Biofilms were grown for 3 days continuous flowing conditions in tube reactors and then exposed to tobramycin (150 μg/ml) for 1 h under flowing conditions. Biofilms formed the by *P. aeruginosa* wild-type strain PAO1 harboring the empty vector pMJT-1 were used as controls. Viability was determined via CFU counts. Susceptibility is expressed as log_10_ reduction in CFU counts. Experiments were performed using biological triplicates. Error bars indicate standard deviations. *significantly different from PAO1/pMJT-1, *p* < 0.05, as determined by ANOVA followed by a Dunnett’s post-hoc test. **f** Representative confocal images of biofilms formed by ∆*sagS* expressing homologs of PA2146 from *E. coli* (*ymdF*_Ec_) and *K. pneumoniae* (*ymdF1*_*Kp*_). Biofilms were grown for 5 days under flowing conditions. Prior to confocal imaging, biofilms were stained with *Bac*Light LIVE/DEAD-stain. **g** Antimicrobial susceptibility of biofilms formed by ∆*sagS* harboring an empty vector or expressing homologs of PA2146 from *E. coli* (*ymdF_EC*) and *K. pneumoniae* (*ymdF1_KP, ymdF2_KP, ymdF3_KP*). Biofilms were grown for 3 days continuous flowing conditions in tube reactors and then exposed to tobramycin (150 μg/ml) for 1 h under flowing conditions. Biofilms formed the by *P. aeruginosa* wild-type strain PAO1 harboring the empty vector pMJT-1 were used as controls. Viability was determined via CFU counts. Susceptibility is expressed as log_10_ reduction in CFU counts. Experiments were performed using biological triplicates. Error bars indicate standard deviations. *, significantly different from PAO1/pMJT-1, *p* < 0.05, as determined by ANOVA followed by a Dunnett’s post-hoc test. **h**, **i** Detection of BrlR abundance in biofilms formed by ∆*sagS* strains harboring a chromosomally encoded V5/His_6_-tagged BrlR under the control of its own promoter (P_*brlR*_*-brlR-*V5/His_6_), by immunoblot analysis. Biofilms by *P. aeruginosa* PAO1 harboring a chromosomally encoded V5/His_6_-tagged BrlR under the control of its own promoter (P_*brlR*_*-brlR-*V5/His_6_) and the empty vector pMJT-1 was used as control. The immunoblots (IBs) were probed for the presence of BrlR using anti-V5 antibodies. Lower panel shows Coomassie-stained TCE used as controls for the immunoblot analysis. All experiments were performed in triplicate. Representative images are shown. Blots and gels were derived from the same experiment. Experiments were performed in triplicate and representative images are shown. Images of the uncropped blots are shown in the supplementary material. **h** Immunoblot analysis of total cell extracts (TCE) from 3-day biofilms of *P. aeruginosa* PAO1/pMJT-1 or Δ*sagS* bearing pMJT-1 or expressing *sagS*, PA2146, or the *E. coli* homolog of PA2146, *ymdF*_*Ec*_. **i** Immunoblot (analysis of total cell extracts (TCE) from 3-day biofilms of *P. aeruginosa* PAO1/pMJT-1 or Δ*sagS* bearing pMJT-1 or expressing *sagS*, PA2146, or one of the three *K. pneumoniae* homologs of PA2146 (*ymdF1*_*KP*,_ *ymdF1*_*KP*,_ or *ymdF1*_*KP*_).

Our findings so far suggested PA2146 to be highly expressed in biofilms formed by *P. aeruginosa, E. coli and K. pneumonia, w*ith insertional inactivation of PA2146 and its homologs coinciding with the formation of thin and unstructured biofilms with increased susceptibility to antimicrobial agents. As c-di-GMP has been linked to the biofilm formation and biofilm susceptibility^68–70^, we next asked whether biofilms by PA2146 mutants are characterized by reduced cellular c-di-GMP levels. We made use of an unstable GFP reporter (PcdrA::*gfp*(ASV)) for which the fluorescence intensity is directly proportional to the concentration of intracellular c-di-GMP^71^. No difference in cellular c-di-GMP levels were noted between wild-type and ∆PA2146 grown planktonically to exponential phase (Fig. 8a). In contrast, biofilms formed by ∆PA2146 following growth in 24-well plates for 3 days were found to harbor reduced cellular levels of c-di-GMP relative to wild-type biofilms (Fig. 8a). Multicopy expression of PA2146 in ∆PA2146 restored c-di-GMP levels to wild-type levels (Fig. 8a). C-di-GMP levels were furthermore quantitated using an HPLC-based assay. Biofilms formed by ∆PA2146 under flowing conditions were found to harbor reduced cellular levels of c-di-GMP relative to wild-type biofilms (Fig. 8b), corroborating the unstable GFP reporter-based results of ∆PA2146 biofilms harboring reduced c-di-GMP levels relative to wild-type biofilms.

It is of interest to note that the phenotype of ∆PA2146 is reminiscent of *P. aeruginosa* ∆*sagS* biofilms. *sagS* encodes the orphan sensor SagS, that has previously been reported to promote the switch from planktonic to biofilm growth, likely via phospho-signaling to the BfiSR regulatory system, apparent by biofilms formed by sagS being flat and unstructured^26,57^, and to be required for biofilm drug tolerance, with *sagS* inactivation correlating with significantly reduced the expression of a gene encoding the MerR-like transcriptional regulator BrlR^57,58,72^. BrlR is a global regulator of biofilm resistance that modulates the tolerance to a broad range of antimicrobial agents in *P. aeruginosa* biofilms, and in turn, reduced expression of genes encoding multidrug efflux pumps and ABC transporters^73–75^. Moreover, biofilms by ∆*sagS* harbor reduced cellular levels of c-di-GMP relative to wild-type biofilms^57,58,75^.

Restoration of c-di-GMP levels by overexpression of *gcbA*, encoding the diguanylate cyclase GcbA, restored the three-dimensional architecture of ∆PA2146 biofilms to wild-type levels (Fig. 8c). Overexpression of *bfiS*, encoding the two-component sensor and SagS-interacting partner BfiS^26,76^ (Fig. 8c), likewise restored the architecture of ∆PA2146 biofilms to wild-type levels.

The similarity in phenotypes of biofilms formed by ∆PA2146 and ∆*sagS* suggested PA2146 and SagS to likely play very similar roles in biofilm developmental processes. We therefore asked whether SagS and PA2146 work in concert. Overexpression of *sagS* in ∆PA2146 failed to rescue the mutant phenotypes, as evidenced by ∆PA2146/pMJT-*sagS* exhibiting impaired biofilm development and elevated susceptibility to tobramycin similar to the trends observed for ∆PA2146/pMJT-1 (Fig. 8d, e). In contrast, multi-copy expression of PA2146 in *ΔsagS* restored biofilm formation to wild-type levels, while also rescuing the susceptibility phenotype, apparent by tobramycin treatment reducing the viability of *ΔsagS/*pMJT-PA2146 biofilm cells by only 0.8 log CFU (Fig. 8d, e).

We furthermore asked whether *K. pneumoniae ymdF* inparalogs or *E. coli ymdF* are capable of restoring *ΔsagS* biofilm phenotypes to wild-type levels. Similar to PA2146, multicopy expression of *K. pneumoniae ymdF* inparalogs or *E. coli ymdF* restored architecture and susceptibility to tobramycin of *ΔsagS* biofilms to wild-type levels (Fig. 8f, g, Table 3). Taken together, these data suggested that PA2146 likely functions downstream of SagS.

SagS has been previously reported to contribute to the abundance of BrlR, a c-di-GMP-responsive transcriptional regulator, that activates the expression of several multidrug efflux pumps and ABC transporters, to confer drug tolerance of biofilms^25,26,58,73–77^. Considering that PA2146 likely functions downstream of SagS, we therefore asked whether PA2146 contributes to BrlR abundance. In agreement with previous findings^58,72^, little to no BrlR was produced by *ΔsagS* biofilms relative to wild-type biofilms while multi-copy expression of PA2146 in *ΔsagS* restored BrlR production to wild-type levels (Fig. 8h). Moreover, BrlR abundance in *ΔsagS*/pMJT-PA2146 biofilms was comparable to that noted in cell extracts obtained from *ΔsagS*/pMJT-*sagS* biofilms. The findings suggest that in a manner similar to SagS, PA2146 contributes to the abundance of BrlR in *P. aeruginosa* biofilms. We also explored whether PA2146 homologs are capable of substituting for PA2146 by contributing to BrlR abundance. Immunoblot analysis demonstrated the presence of BrlR in cross-complemented strains including sagS overexpressing the *E. coli ymdF* and *K. pneumoniae ymdF* inparalogs (Fig. 8i). Our findings strongly suggested that the PA2146 homologs by *P. aeruginosa, E. coli* and *K. pneumoniae* are not only conserved in sequence but also in function.

## Discussion

The goal of the current research was to determine if hypothetical and previously uncharacterized genetic determinants contribute to biofilm formation. We reasoned that by focusing on hypothetical genes or those not previously linked to biofilm formation by *P. aeruginosa*, that we will identify far uncharacterized proteins that may play critical roles in the development of biofilms and biofilm ecology, as well as gain further insight into what is required for biofilm formation, but potentially identify factors unique to biofilms. To identify such genetic determinants, we made use of the biofilm model organism *P. aeruginosa* and carried out a comparative analysis of the biofilm transcriptome using RNA-seq, selected 27 hypothetical and uncharacterized genes that were induced upon transition to the surface associated mode, and evaluated mutants in genes of interest for two biofilm characteristics, biofilm architecture and biofilm drug tolerance.

Our screen revealed 12 out of 27 genes to contribute to the formation of structured biofilms (Table 1). Our findings are in agreement with recent finding of uncharacterized biofilm factors contributing to biofilm formation. Willet et al.^19^ screened an arrayed transposon (Tn) library containing ~2000 *Enterococcus faecalis* mutants in hypothetical genes/intergenic regions and identified eight uncharacterized predicted protein-coding genes required for both early and late biofilms, with OG1RF_10435 harboring phosphatase activity being required for surface attachment, and OG1RF_10435 leading to widespread differences in protein expression and altered arginine catabolism. Likewise, Moorthy and Watnick^18^ inferred the expression of previously uncharacterized biofilm-related genes to virulence gene expression and anaerobic respiration.

Our screen furthermore revealed that 13 out of the 27 genes contributed to the susceptibility phenotype of *P. aeruginosa* biofilms. However, the majority of the mutant biofilms were susceptible to only one of the two antimicrobial agents tested. Notable exceptions are biofilms formed by transposon mutants PA2146::IS and PA2184::IS which were found to be susceptible to both tobramycin and hydrogen peroxide (Table 1). It is of interest to note that both PA2146 and PA2184 are located on the genome in close proximity to *katE* and *katN*^78^, respectively, with STRING predicting PA2146 to interact with both catalase KatE, as well as PA2184 (Fig. 3). However, no such associations are apparent for the other factors contributing to the susceptibility of mutant biofilms to hydrogen peroxide (Fig. 3, Table 1). Whether PA2146 and PA2184 directly or indirectly affect the expression of *katE* and *katN* and/or modulate the activity of catalases KatE and KatN will be the subject of future studies. Additionally, only 6 of the 27 genes affected both susceptibility and the formation of structured biofilms (Table 1). These findings support the notion that susceptibility to antimicrobial agents does not correlate with altered biofilm architecture. This is further supported by the finding that while susceptible to both tobramycin and hydrogen peroxide, PA2184::IS formed wild-type like biofilms while biofilm formation by PA2146::IS (and ∆PA2146) appeared to be impaired (Figs. 1–2, Table 1).

Amongst the factors identified in this study to affect biofilm formation and/or biofilm susceptibility, we chose to focus on PA2146 encoding a small 5 kDa protein for various reasons. For one, inactivation of PA2146 affected the formation of structured biofilms as well as rendered the mutant biofilms susceptible to both tobramycin and hydrogen peroxide. Moreover, the phenotype of biofilms formed by ∆PA2146 was reminiscent of that of ∆*sagS*, with homologs of PA2146 having been described in Gram-negative and Gram-positive bacterial species to play a role in stress response. Here, however, we were unable to link PA2146 to stress other than to the mode of biofilm growth. While biofilm formation has been linked to various stressors^13^, with PA2146 being induced in response to biofilm-specific (and likely stressful) conditions, it is also likely that PA2146 not contributing to stressors tested here may be due to PA2146 not harboring a complete KGG repeat motif. According to Pfam (https://pfam.xfam.org/family/KGG), the KGG repeat motif (PF10685) contains a highly conserved, characteristic sequence motif, KGG, followed by a Walker nucleotide binding motif GXXXKF(S,T), that in YciG of *E. coli* is QSGGNKSGKS (Fig. 4a, Supplementary Fig. 6b,c). Interestingly, while PA2146 harbors KGG, it lacks the downstream Walker A motif (Fig. 4a, Supplementary Fig. 6b,c). Our analysis revealed that PA2146 was not only found to be highly conserved in the genomes of various bacterial species, but PA2146 and its homologs exhibited some of the most substantial increases in transcript abundance during *P. aeruginosa*, *E. coli*, and *K. pneumoniae* biofilm growth (Tables 1–2, Supplementary Tables 1, 3). Moreover, similar to inactivation of PA2146, inactivation of its homologs impaired biofilm development and affected susceptibility to antimicrobial agents across species (Fig. 5). The findings suggested PA2146 to likely play similar roles in biofilm formation/susceptibility in diverse bacteria. This was further supported by the *K. pneumoniae* and *E. coli* homologs of PA2146 rescuing both development and antimicrobial susceptibility of *P. aeruginosa* ∆PA2146 biofilms to wild-type levels (Fig. 5). Likewise, PA2146 and its homologs restored biofilm formation, antimicrobial susceptibility, and BrlR production by ∆*sagS* mutant biofilms to wild-type levels (Figs. 6, 8). Our findings thus suggest a high degree of sequence and functional conservation for this family of PA2146 homologs. Moreover, our findings strongly suggest that our screen resulted in the identification of a conserved genes that exhibit similar biofilm-specific expression patterns in three ɣ-proteobacterial species.

The identification of a conserved biofilm-associated gene may have several implications such a gene being biofilm ‘founder’ or ‘inventor’ genes or representing a distinct fingerprint gene of biofilms, which Karatan and Watnick^9^ defined as a set of physiological and genetic parameters common to all biofilms. Several transcriptomic and proteomic studies have attempted to identify fingerprint genes. While these studies have shed light on some common trends linked to biofilm formation including repression of flagellar gene expression, upregulation of matrix synthesis gene expression, and upregulation of genes involved in adaptation to stationary phase, environmental stress, and anaerobiosis (reviewed in^10,11,13^), they have not produced a unique biofilm fingerprint. Not surprisingly, Karatan and Watnick^9^ thus concluded that while biofilm cells are distinct from planktonic cells and that certain traits are more common in biofilm-associated cells than in planktonic cells, there is no proteomic, transcriptomic, or matrix analysis that uniquely defines a fingerprint that can be used to characterize a bacterial assemblage as a biofilm. We do not believe that PA2146 is such a fingerprint because it is not conserved in all prokaryotes. However, PA2146 is present in many Gram-negative and Gram-positive species, suggesting that it may have been subjected to adaptation and/or horizontal gene transfer. Moreover, PA2146 was found to be completely identical to a homolog in *Pseudomonas fluorescens* strain NCTC10783 (GenBank LR134300), which supports the view that acquisition of this gene by horizontal transfer may occur readily. Such transfers, and the fact that many taxa of *Pseudomonas* and *Enterobacteriaceae* carry two or more divergent gene loci homologous to PA2146 (Fig. S6), imply that the evolutionary history of this gene has been more complicated than a simple process of vertical descent mirroring organism phylogeny. Related species pairs such as *Enterobacter asburiae*/*Enterobacter cloacae* have retained the same two pairs of related PA2146 homologs (Fig. S6), which suggests that in some cases there has been long-term evolutionary maintenance of multiple copies of this gene. The finding of a well-supported clade of eight *Pseudomonas* species carrying homologs closely related to PA2146 (upper lineage in Fig. S6) likewise suggests that this gene has been functionally important enough to be conserved across taxa adapted to diverse environments. Since this set of *Pseudomonas* PA2146 homologs are all more similar to one another than to any Enterobacteriaceae sequences, it appears that there has been a relatively long period of separate evolution of this gene within *Pseudomonas* after its initial acquisition from some type of enterobacterial ancestor.

While it is apparent that PA2146 homologs are not conserved in all prokaryotes, PA2146 and its homologs nevertheless represent a family of biofilm-associated genes that are highly conserved throughout diverse bacterial species, particularly in ɣ-proteobacterial species inhabiting a large range of environments and differing in biofilm-associated functions including cell communication, biofilm matrix production, and motility.

## Methods

Bacterial strains, media, and culture conditions. *Pseudomonas aeruginosa* PAO1, *Escherichia coli* BW25113, and *Klebsiella pneumoniae* MKP103, as well as isogenic mutants in the respective parental backgrounds, were used as indicated and are listed in Supplementary Table 4. The PAO1 transposon mutants were obtained from the sequence-verified two-allele library^79^, the *K. pneumoniae* mutants from the transposon mutant library of *K. pneumoniae* outbreak strain KPNIH1^80^, and the *E. coli* mutant originated from the *E. coli* K-12 in-frame, single-gene knockout mutant collection^81^. All planktonic cultures were grown in Lennox Broth (LB, BD Biosciences) in flasks at 220 rpm and 37 °C. Biofilms were grown as indicated below. Antibiotics for plasmid maintenance were used at the following concentrations: 250 μg/ml carbenicillin and 50 to 75 μg/ml gentamicin for *P. aeruginosa* and 100 μg/ml ampicillin and 20 μg/ml gentamicin for *E. coli*.

### Strain construction

The PA2146 isogenic mutant was constructed by allelic replacement using sucrose counterselection enabled by the gene replacement vector pEX18Gm^82^. Specifically, the regions flanking PA2146 were amplified using primers listed in Supplementary Table 5 and cloned sequentially into pEX18Gm to generate the gene replacement vector pEX18-PA2146, which was subsequently introduced into *P. aeruginosa* PAO1 via conjugation. Unmarked double-crossover mutants were isolated by sucrose-mediated counter-selection in the presence of 7.5% sucrose and confirmed by PCR and sequencing. The genes PA2146 and *rsmA* from *P. aeruginosa*, *ymdF* and *csrA* from *E. coli*, and the *K. pneumoniae ymdF* inparalogs (KPNIH1_09545, KPNIH1_10100, and KPNIH1_13915) were amplified using primers listed in Supplementary Table 5 from *P. aeruginosa* PAO1, *E. coli* BW25113, or *K. pneumoniae* MKP103 genomic DNA and placed under the control of an arabinose-inducible P_BAD_ promoter in the pMJT-1 vector^83^. PA2146 and PA2184 were cloned into the bacterial two-hybrid vectors pKT25 and pUT18c by ligation. All plasmids were introduced via electroporation. Plasmid inserts were verified by DNA sequencing. For monitoring transcription from the *P. aeruginosa* PA2146 promoter, luciferase promoter fusion constructs were created by cloning the putative promoter region of *ymdF*_Pa_ (located −1 to −513 bp upstream of the *ymdF*_Pa_ start codon) into mini-CTX-*lux* to create the vector CTX-PA2146-*lux*, which was then introduced into PAO1 via conjugation^84^. The vector backbone and Tet^R^ marker was subjected to Flp-mediated excision via electroporation of the plasmid pFLP2^85^ to create the unmarked strain PAO1::*P*_*PA2146*_*513-lux*. A promoterless control *lux* strain, PAO1::*lux*,was created by introducing the empty mini-CTX-*lux* vector into PAO1.

### Luminescence reporter assays

PAO1::*P*_*PA2146*_*513-lux* and PAO1::*lux* strains grown to mid-exponential or stationary phase as planktonic cells, in 24-well plates for 6 h for initial attachment, or as biofilms in tube reactors for 3 or 6 days were adjusted to OD600 ~0.25. Subsequently, the optical density at 600 nm and luminescence were read using a SpectraMax i3x plate reader (Molecular Devices), with the default luminescence read settings. The luminescence readings were first adjusted to OD600, followed by subtraction of the adjusted PAO1::*lux* promoterless *lux* negative control from the respective adjusted readings for PAO1::*P*_*PA2146*_*513-lux*.

### Attachment

Attachment to a polystyrene surface was measured using the 96-well plate assay with crystal violet staining following 24 h of growth in LB medium, with the plates incubated at 220 rpm to ensure proper aeration and prevent cell lysis^86^. An inoculum (200 μl) having an optical density at 600 nm (OD600) 0.025 was used.

### Biofilm growth

For antimicrobial susceptibility testing, biofilms were grown using a continuous flow tube reactor system (1 m long size 13 silicone tubing, Masterflex, Cole Parmer, Inc.) with an inner surface area of 25 cm^2^ at a flow rate of 0.1 ml/min^87,88^. *P. aeruginosa* and *E. coli* were grown in 5% LB medium and *K. pneumoniae* was grown in 10% MOPS-glucose medium (100% MOPS-glucose medium: 50 mM MOPS sodium salt, 93 mM NH_4_Cl, 43 mM NaCl, 2 mM KH_2_PO_4_, 1 mM MgSO_4_, 30 mM glucose, 0.1 mg/L CuSO_4_·5H_2_O, 0.1 mg/L ZnSO_4_·H_2_O, 0.1 mg/L FeSO_4_·7H_2_O, 0.004 mg/L MnCl_2_·4H_2_O, pH 7.2). Following 3–6 days of growth, the biofilms were subjected susceptibility testing. For the analysis of the biofilm architecture, *K. pneumoniae* biofilms were grown in 96-well plates (not-treated polystyrene surface) to promote attachment in MOPS-glucose medium for up to 6 days. *E. coli* biofilms were grown under flowing conditions using flow cell reactors (glass surface, BioSurface Technologies) in 5% LB medium at a flow rate of 0.2 ml/min) for up to 6 days, while *P. aeruginosa* biofilms were grown either 24-well plates (polystyrene, not-treated) as well as flow cell reactors using 5% LB medium at a flow rate of 0.2 ml/min for up to 6 days. For microtiter plate grown biofilms, the medium was exchanged every 12 h. Following 3–6 days of growth, the biofilms were subjected to microscopy analysis.

### Growth conditions for RNA-seq

For RNA seq, biofilms by *P. aeruginosa* were grown in 20-fold diluted LB medium using a continuous flow tube reactor system as described above at a flow rate of 0.1 ml/min for 3 days. Following 3 days of growth, the biofilms were harvested by pinching the tubing, which resulted in the biofilm biomass being extruded from the inner surface of the tube reactor. Biofilm cells were sampled directly into 3 mL of RNAprotect Bacteria Reagent (Qiagen) as described below. Planktonic cells were grown in LB to exponential phase in flasks with shaking at 220 rpm and 37 °C, and subsequently collected into RNAprotect Bacteria Reagent as described below.

### RNA isolation, processing, and analysis

Samples of planktonic or biofilm *E. coli*, *P. aeruginosa*, or *K. pneumoniae* cells were collected into 3 mL of RNAprotect Bacteria Reagent (Qiagen). Following incubation for 10 min at room temperature, mRNA isolation (RNeasy Mini Kit, Qiagen) was carried out using approximately 3 × 10^8^ cells according to the manufacturer’s protocol, with the following modifications. The cells were treated with 400 µg/mL lysozyme in TE (10 mM Tris-HCl, 1 mM EDTA, pH 8.0) for 5 min at room temperature prior to RNeasy RNA isolation. The RNeasy elution step was modified to use 75uL TE to ensure downstream stability of the RNA during the RNA-seq library preparation. For RNA-seq analysis, the RNA was subsequently subjected to DNase treatment (TURBO DNA-*free* DNase Treatment, Ambion) according to the manufacturer’s protocol. RNA quantity and quality were assessed as described below.

### RNA and DNA quantity and quality assessment

RNA or DNA was quantified using Qubit RNA HS Assay Kit and Qubit dsDNA HS Assay Kit (Life Technologies), respectively, on the Qubit 2.0 Fluorometer (Life Technologies). Fragment size distribution of RNA or DNA samples was assessed on the Agilent 2100 BioAnalyzer using the Agilent RNA 6000 Pico Kit or Agilent High Sensitivity DNA Kit (Agilent Technologies), respectively. The BioAnalyzer was also used to assess the quality of DNAse-treated total RNA, with only RNA samples exhibiting an RNA integrity number (RIN) > 9 selected for downstream processing^89^.

### RNA-seq library construction and sequencing

Whole transcriptome RNA-seq of PAO1 planktonic and biofilm cells was carried out using the ION Torrent Personal Genome System (Life Technologies)^90^. Following depletion of rRNA using RiboZero (illumina), the cDNA libraries were constructed using the Ion Total RNA-Seq kit v2 (Life technologies) according to the manufacturer’s protocol. The cDNA libraries were used to prepare sequencing templates using the Ion PGM Template OT2 200 kit on the Ion OneTouch™2 instrument (Life Technologies), followed by Ion Sphere™ Quality Control Kit assessment and template-positive Ion Sphere™ Particle (ISP) enrichment using the Ion OneTouch™ ES instrument. Sequencing was carried out using the Ion Sequencing 200 Kit v2 and the Ion 316v2 Chip on the Ion Torrent PGM sequencing platform (Life Technologies). RNA-seq was performed in triplicate using biological replicates. The raw sequencing data are available in the NCBI short read archive (http://www.ncbi.nlm.nih.gov/Traces/sra/sra.cgi) with accession number SRP096901 under BioProject PRJNA362216.

### RNA-seq data analysis

FastQC (Babraham Bioinformatics) was used to generate statistics and evaluate the quality of the generated RNA-seq reads (see [http://www.bioinformatics.bbsrc.ac.uk/projects/fastqc/]). Bowtie 2^91^ with default parameters was applied to map the RNA-seq reads to the *P. aeruginosa* PAO1 reference genome^32^, and SAMtools were used to convert the reads to a BAM format for downstream analysis^92^. Qualimap was then used to generate alignment statistics and to compute the number of counts per genomics element^93^. In addition, biotype distribution and depth of coverage analyses were performed using the R package NOISeq^94^. Differential gene expression analysis, including significance calculations, was carried out using the R package edgeR^95^. All RNA-seq related procedures were carried out in triplicate using biological replicates.

### Quantitative reverse-transcription PCR (qRT-PCR)

cDNA synthesis was performed using the iScript™ cDNA Synthesis Kit (BioRad), with 1 μg of total RNA used as the template. Subsequently, qRT-PCR for indicated transcripts was performed using the BioRad CFX Connect Real-Time PCR Detection System and SsoAdvanced™ SYBR® Green Supermix (BioRad) with oligonucleotides listed in Table S5. For relative quantitation of transcript abundance, the CFX Manager Software (BioRad) was used to first normalize transcript abundance (based on the threshold cycle value (Ct)) to *mreB* (*P. aeruginosa* and *E. coli*) or *rpoB* (*K. pneumoniae*) and then to determine the transcript abundance ratios. Melting curve analyses were employed to verify specific single product amplification. The qRT-PCR analysis was performed using biological triplicates.

### Immunoblot analysis and pulldowns

While V5-tagged YmdF_Pa_ was undetectable by direct immunoblotting of total cell extracts, the protein was detected following enrichment using a preliminary immunoprecipitation procedure. Briefly, V5-tagged YmdF_Pa_ was immunoprecipitated from 200 μg of total cellular protein extracts using anti-V5 antibodies (1 μg/mL) immobilized on protein A/G agarose beads (Cell Signaling). The immunoprecipitation eluates were then subjected to immunoblotting analysis as described below. Additionally, the abundance of V5-tagged BrlR in ∆*sagS* strains overexpressing PA2146 and various homologs was assessed by SDS-PAGE and immunoblotting. Total cell extracts (30 µg per sample) were resolved on a 15% polyacrylamide gel and subsequently transferred onto a polyvinylidene difluoride (PVDF) membrane using a TurboTransblot apparatus (Bio-Rad). Western blots were probed with at 0.1 μg/mL anti-V5 antibodies (Life Technologies) and developed using the Immun-Star WesternC chemiluminescence kit (Bio-Rad). Following transfer, SDS-PAGE gels were Coomassie stained to ensure equal loading.

Pull-down assays were used to assess the interactions between PA2146 and PA2184 in total protein cell extracts of cells co-producing V5-tagged PA2146 and HA-tagged PA2184 protein. Following immunoprecipitation of V5-tagged proteins using immobilized anti-V5 antibodies at a 2 µg/mL concentration, immunoprecipitation eluates were separated by SDS/PAGE and assessed by immunoblot analysis for the presence of HA-tagged PA2184 proteins using anti-HA antibodies as described above. Pull-down assays were carried out using 200 μg protein from cellular extracts.

### Biofilm architecture analysis

Architecture of biofilms grown in flow cells or in microtiter plates was assessed via confocal laser scanning microscopy (CLSM). CSLM was carried out using a Leica TCS SP5 confocal microscope. Prior to confocal microscopy, biofilms were stained using the *Bac*Light LIVE/DEAD viability stain (Life Technologies) at a 1/1000 dilution in the growth medium. The CLSM images were processed using LAS AF software v2.4.1. Quantitative analysis of the images was performed using the COMSTAT^96^. For brightfield visualization of biofilm architecture, the samples were viewed by transmitted light using an Olympus BX60 microscope and Olympus UPLNFLN 20× and 40× objectives. Images were captured using a ProgRes CF camera (Jenoptik, Jena, Thuringia) and processed using ProgRes CapturePro 2.7.7 software.

### Biofilm antibiotic susceptibility assays

All biofilms subjected to antimicrobial susceptibility assays were grown in tube reactors. However, due to the differences in biomass accumulation and innate susceptibility to antimicrobial agents, the duration of growth and antimicrobial concentrations differed for the various bacterial species. Biofilms grown for 3 days (*P. aeruginosa* and *K. pneumoniae*) or 4 days (*E. coli*) were treated for 1 h under continuous flowing conditions with tobramycin (75 or 150 μg/mL for *E. coli* or *P. aeruginosa*, respectively), ciprofloxacin (150 μg/mL for *E. coli*), gentamicin (100 μg/mL for *K. pneumoniae*), or colistin (300 μg/mL for *K. pneumoniae*). Following exposure of biofilms to the respective antimicrobial agents under flowing conditions, biofilms were harvested from tube reactors by squeezing the tubing, followed by the extrusion of the cell paste^87^. Cells exposed to antimicrobial agents were either washed (1 min at 16000 × g) twice with 1 mL saline prior to dilution or as in the case of ciprofloxacin and hydrogen peroxide, diluted into saline containing 10 mM magnesium chloride or 100 mM sodium thiosulfate to inactivate the respective antimicrobial agent. To ensure complete disaggregation of cell aggregates, the resulting suspension was homogenized for 15 sec using a tissue tearor (Biospec). The cell suspension was then serially diluted and spread plated onto LB agar. Viability was determined via CFU counts. Susceptibility is expressed as log_10_ reduction in viability.

### Minimum inhibitory concentrations (MICs)

MICs of tobramycin or ciprofloxacin were determined using 2-fold serial dilutions in LB medium in 96-well microtiter plates. Tobramycin and ciprofloxacin concentrations ranged from 0.05 to 50 μg/ml and 0.0078 to 4 μg/ml, respectively. Following inoculation of approximately 10^4^ cells per well, the microtiter plates were incubated for 24 h at 37°C and 220 rpm. OD600 was used to determine the MIC, defined as the lowest antibiotic concentration that resulted in no detectable bacterial growth.

### Phylogenetic tree construction

For phylogenetic trees demonstrating the evolutionary relationships for PA2146/*ymdF* homologs, BLAST searches were performed using both nucleotide and amino acid sequences of PA2146 to identify related sequences. Sequences were aligned using CLUSTALW. Phylogenetic trees were inferred using maximum likelihood (ML), neighbor-joining (NJ), and Bayesian algorithms using MEGA7 and MrBayes3.2.2 software. Branch support was inferred by bootstrapping (ML, NJ) or from posterior probability values (Bayesian analysis).

### Bacterial adenylate cyclase two-hybrid assays

Protein-protein interactions were assessed by the bacterial adenylate cyclase two-hybrid (BACTH)^97,98^. Briefly, proteins of interest were fused to the T18 or T25 fragment of *Bordetella pertussis* adenylate cyclase. T18 and T25 fusion proteins were then coexpressed in *E. coli* DHM1 to test for interaction. Interaction between the two hybrid proteins reconstitutes the catalytic domain of adenylate cyclase, leading to cyclic AMP (cAMP) synthesis and transcription of the *lac* operon. DHM1 transformants were OD-adjusted, 10-fold serially diluted and 2 µl per dilution spotted onto LB agar containing 50 µg/ml ampicillin, 50 µg/ml kanamycin, X-Gal (40 µg/ml), and IPTG (isopropyl-β-D-thiogalactopyranoside, 0.1 mM). Plates were incubated at 30 °C for up to 72 h, and colonies were examined for blue coloration. TorR and TorS protein served as positive controls^33^, and the empty plasmids acted as the negative control. The efficiencies of these interactions were quantified using β-galactosidase activity assays^99^.

### Quantification of c-di-GMP

Cyclic di-GMP was extracted in triplicate from wild-type and mutant strains using heat and ethanol precipitation^100^ and quantitated using an Agilent 1100 HPLC (flow rate of 0.2 ml/min, detector set to 253 nm, reverse-phase C_18_ Targa column (2.1 × 40 mm; 5 µm))^101,102^. Commercially available cyclic di-GMP was used as a reference for the identification and quantification of cyclic di-GMP in cell extracts. In addition, relative c-di-GMP levels of 3-day old biofilms grown under flowing conditions in tube reactors, were determined using a fluorescence-based assay that takes advantage of the c-di-GMP responsive *cdrA* promoter fused to unstable GFP (P*cdrA*::gfp(ASV))^71^. Biofilms were grown 3 days in 24-well plates as described above, plates were washed with 0.85% saline to remove planktonic cells. Biofilms were then harvested by scraping into 0.85% saline, and the resulting suspension homogenized to ensure disaggregation. Planktonic cells were grown to exponential phase and washed using saline. The absorbance (600 nm) and fluorescence (GFP: 485/535 nm; nm) of planktonic and biofilm cells was measured in a 96-well black clear-bottom microtiter plate (Greiner Bio-One) using a SpectraMax i3x plate reader (Molecular Devices). To ensure correlation between absorbance (600 nm) and fluorescence, measurements were also taken of serially 2-fold diluted samples. Quantifications were performed in triplicate using biological replicates, and the fluorescence unit from GFP was normalized to absorbance.

### Statistical analysis

For pairwise comparison, a two-tailed Student’s t-test assuming equal variance or using single-factor analysis of variance (ANOVA), followed by a Dunnett’s post-hoc test using Prism5 software (Graph Pad, La Jolla, CA, USA), was used. Unless otherwise noted, all experiments were performed at least in triplicate using biological replicates.

## Supplementary information

Supplementary material

## Data Availability

The authors declare that the data supporting the findings of this study are available within the paper and its Supplementary Files. Additional data are available from the corresponding author upon reasonable request.
