# Supplementary material for: A previously uncharacterized gene, PA2146, contributes to biofilm formation and drug tolerance across the ɣ-Proteobacteria
Source: NPJ Biofilms Microbiomes. 2022 Jul 7;8:54. doi: 10.1038/s41522-022-00314-y (PMC9262955; doi:10.1038/s41522-022-00314-y)

SUPPLEMENTARY MATERIALS

A previously uncharacterized gene, PA2146, contributes to biofilm formation and drug tolerance across the ɣ-Proteobacteria

Matthew F. Kaleta, Olga E. Petrova, Claudia Zampaloni, Fernando Garcia-Alcalde, Matthew Parker, and Karin Sauer

- Supplementary Figure legends
- Supplementary Table legends
- Supplementary Figures S1-S7
- Supplementary Table S1-S5
- References
- Original blot images, uncropped

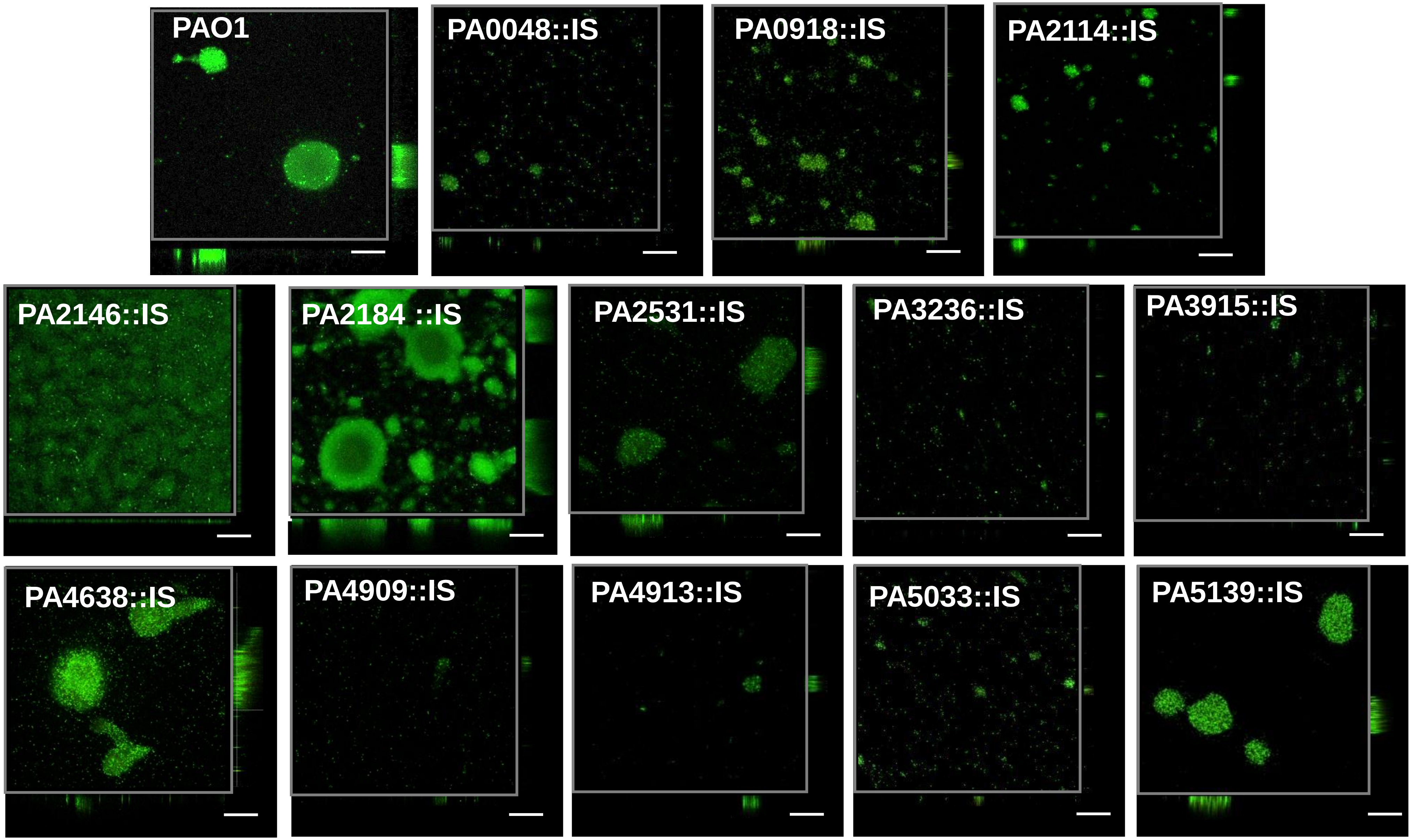
PAO1
PA0048::IS
PA0918::IS
PA2114::IS
PA2146::IS
PA2184 ::IS
PA2531::IS
PA3236::IS
PA3915::IS
PA4638::IS
PA4909::IS
PA4913::IS
PA5033::IS
PA5139::IS

Supplementary Figure 1. Contribution of genes encoding previously uncharacterized proteins to biofilm formation. Representative confocal images of the biofilm architecture of wild type and transposon mutants following 5 days of growth under flowing conditions. *P. aeruginosa* PAO1 was used as control. Biofilms were stained with *Bac*Light LIVE/DEAD stain prior to image acquisition. size bar, 100 µm. All experiments were performed using at least biological duplicates

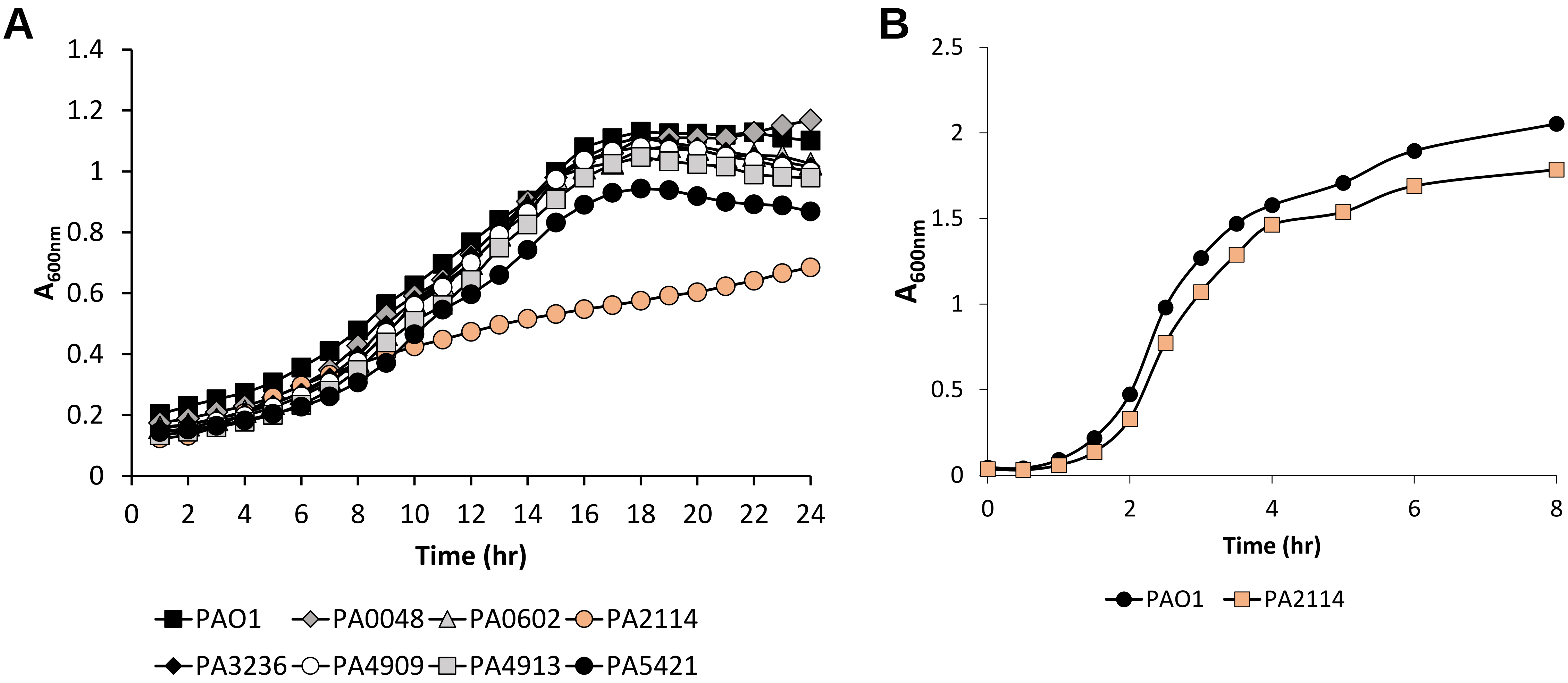
A
B
1.4
1.2
1
0.8
0.6
0.4
0.2
0
A600nm
0 2 4 6 8 10 12 14 16 18 20 22 24
Time (hr)
PAO1
PA0048
PA0602
PA2114
PA3236
PA4909
PA4913
PA5421
2.5
2
1.5
1
0.5
0
0 2 4 6 8
Time (hr)
PAO1
PA2114

Supplementary Figure 2. Growth curves

(A) PAO1 and indicated mutant strains were grown in 96-well plates harboring 200 µL LB per well. Growth was monitored by measuring the absorbance at 600 nm at 37 ^{o}C in a multimode microplate reader (SpectraMax i3x plate reader, Molecular Devices) in 10 minute intervals at which time the cultures were also shalen for 10 seconds. Experiments were carried out in triplicate but only representative growth curves are shown.

(B) As the mutant harboring an insertional inactivation in gene PA2114 demonstrated reduced growth when grown in 96-well plates, grwoth assays were repeated with PA2114::IS and PAO1 grown in Lennox Broth (LB, BD Biosciences) in flasks at 37ºC and continuous shaking at 220 rpm. Absorbance at 600nm was measured using 1 ml aliquots in 30 to 60 minute intervals. Experiments were carried out in triplicate and representative growth curves are shown.

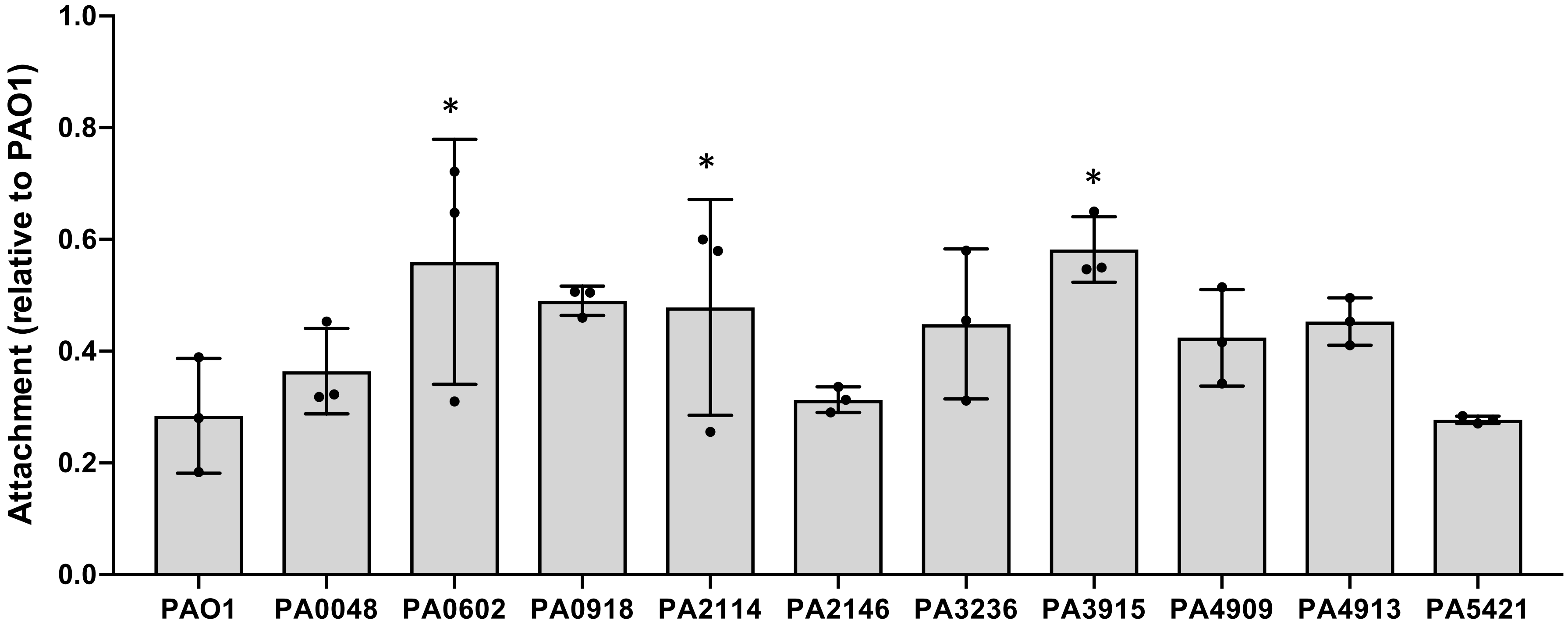

Supplementary Figure 3. Attachment assays.

Attachment by wild-type *P. aeruginosa* PAO1 and mutant strains. Attachment assays were carried out in 96-well plates as previously described using LB medium. Attachment was assessed post 24 hours of incubation using CV staining. Experiments were carried out in duplicate using at least 8 technical replicates each. Error bars indicate standard deviation. [*], statistical significant difference relative to PAO1 (*p*-value < 0.1), as determined using ANOVA followed by a Dunnett's post-hoc test.

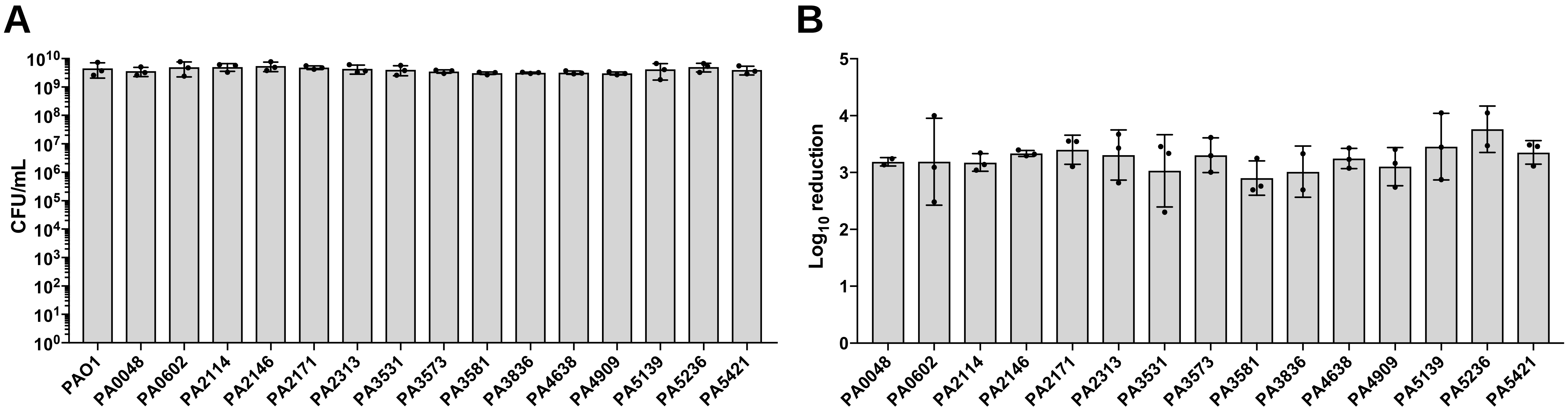

Supplementary Figure 4. Tobramycin susceptibility assays using planktonic cells grown to exponential phase.
PAO1 and indicated mutant strains were grown in LB medium to early exponential phase (A_{600nm}~0.2) in flasks at 37ºC and continuous shaking at 220 rpm. Bacterial suspensions were subsequently exposed to sterile water (untreated) or tobramycin (50 µg/ml) for 30 minutes. Cells exposed to antimicrobial agents were either washed (1 min at 16000 × g) twice with 1 mL saline prior to serial dilution and spread plating onto LB agar. Viability was determined via CFU counts. Susceptibility is expressed as $\log_{10}$ reduction in viability. Experiments were carried out in duplicate using at least 8 technical replicates each. Error bars indicate standard deviation.

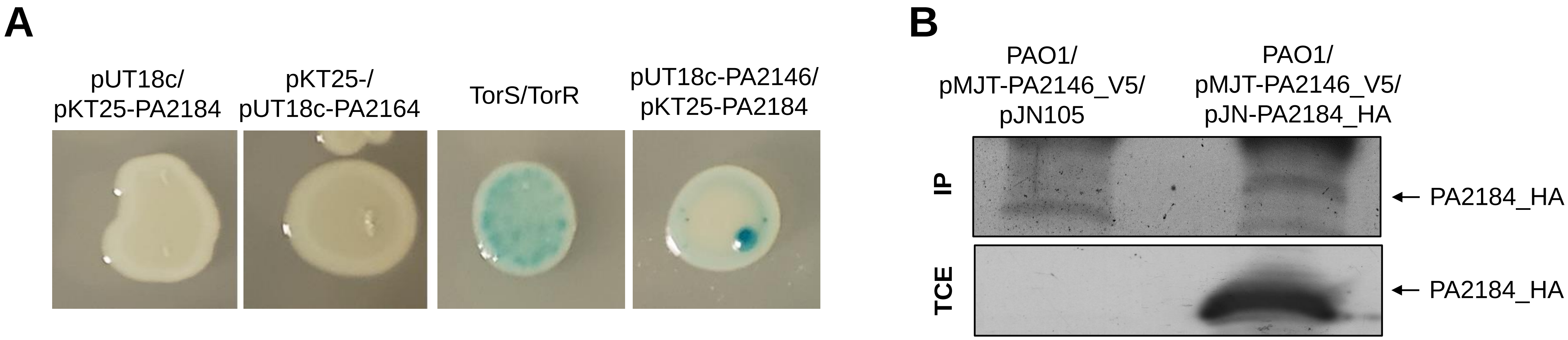

Supplementary Figure 5. Detection of protein interactions.

(A) Representative images of *E. coli* DHM1 cells harboring the plasmids pUT18c-PA2184/pKT25-PA2146. Negative control strains include DHMI/pUT18c/pKT25-PA2146, DHMI/pUT18c-PA2184/pKT25. The positive control harbors plasmids encoding *torR* and *torS*. The respective *E. coli* DHM1 strains were spotted (2 µl) onto LB agar containing ampicillin, kanamycin, IPTG, and X-Gal. Plates were incubated at 30°C for 48 h. Cleavage of X-Gal (blue) indicates a positive protein-protein interaction. Images were obtained from colonies present on the same agar plate. Experiments were performed in triplicates and representative images are shown.

(B) Pull down assay using V5-tagged PA2146 as a bait. Co-purification of HA-tagged PA2184 was detected using immunoblotting with anti-HA antibodies. Data are representative of three independent experiments, all of which gave similar results.

A

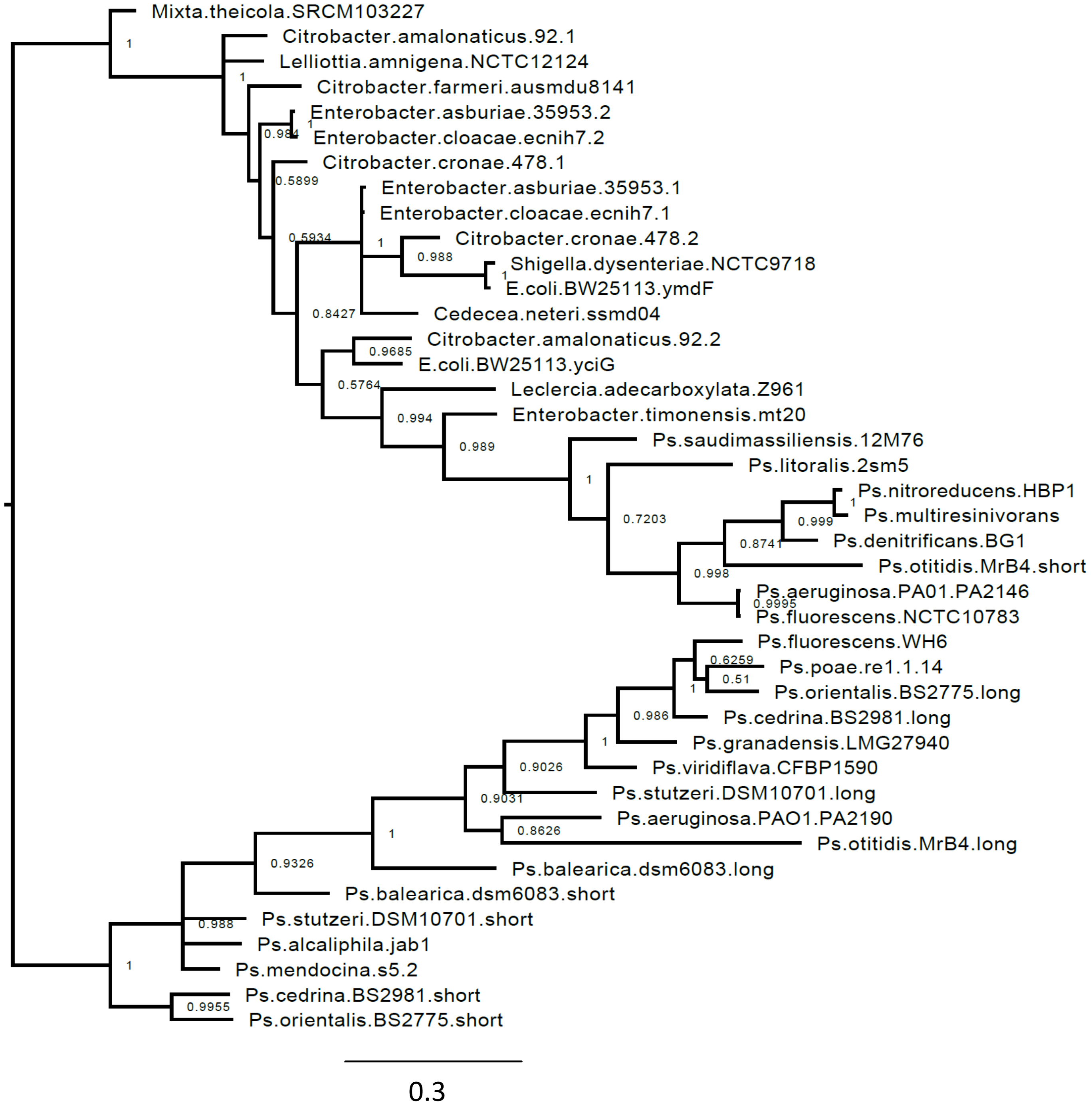
Mixta.theicola.SRCM103227
Citrobacter.amalonaticus.92.1
Lelliottia.amnigena.NCTC12124
Citrobacter.farmeri.ausmdu8141
Enterobacter.asburiae.35953.2
Enterobacter.cloacae.ecnih7.2
Citrobacter.cronae.478.1
Enterobacter.asburiae.35953.1
Enterobacter.cloacae.ecnih7.1
Citrobacter.cronae.478.2
Shigella.dysenteriae.NCTC9718
E.coli.BW25113.ymdF
Cedecea.neteri.ssmd04
Citrobacter.amalonaticus.92.2
E.coli.BW25113.yciG
Leclercia.adecarboxylata.Z961
Enterobacter.timonensis.mt20
Ps.saudimassiliensis.12M76
Ps.litoralis.2sm5
Ps.nitroreducens.HBP1
Ps.multiresinivorans
Ps.denitrificans.BG1
Ps.otitidis.MrB4.short
Ps.aeruginosa.PA01.PA2146
Ps.fluorescens.NCTC10783
Ps.fluorescens.WH6
Ps.poae.re1.1.14
Ps.orientalis.BS2775.long
Ps.cedrina.BS2981.long
Ps.granadensis.LMG27940
Ps.viridiflava.CFBP1590
Ps.stutzeri.DSM10701.long
Ps.aeruginosa.PAO1.PA2190
Ps.otitidis.MrB4.long
Ps.balearica.dsm6083.long
Ps.balearica.dsm6083.short
Ps.stutzeri.DSM10701.short
Ps.alcaliphila.jab1
Ps.mendocina.s5.2
Ps.cedrina.BS2981.short
Ps.orientalis.BS2775.short
1
1
0.984
1
0.5899
0.5934
1
0.988
1
0.8427
0.9685
0.5764
0.994
0.989
1
0.7203
1
0.999
0.8741
0.998
0.9995
0.6259
0.51
1
0.986
1
0.9026
0.9031
0.8626
1
0.9326
0.988
1
0.9955
0.3

B

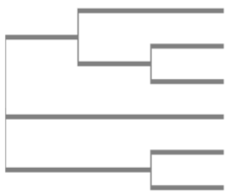

```
PAO1_PA2146              MAQHQGGKGNFAEDPKRASEAGKKGGQASGGNFKNDPQRASEAGKKGGQRSHGGN------ 55
BW25113_ymdF_Ec          MANHRGGSGNFAEDRERASEAGKKGGQHSGGNFKNDPQRASEAGKKGGKSSHGKSDN---- 57
KPNIH1_09545(ymdF1_Kp)   MANHRGGSGNFAEDRERASEAGRKGGQHSGGNFKNDPQRASEAGKKGGKNSHGSRES---- 57
BW25113_yciG_Ec          MAEHRGGSGNFAEDREKASDAGRKGGQHSGGNFKNDPQRASEAGKKGGQQSGGNKSGKS-- 59
KPNIH1_13915(ymdF3_Kp)   MAEHRGGSGNFAEDREKASEAGRKGGQHSGGNFKNDPERASEAGKKGGKNSHGGGRKSGDS 61
KPNIH1_10100(ymdF2_Kp)   MAEHRGGSGNFAEDREKASEAGRKGGQHSGGNFKNDPQRASEAGKKGGQNSHGGGRKSDNS 61
                         **:*:**.****** ::**:**:**** *********:**********: * *
```

C

```
BW25113_YmdF_Ec    1  MANHRGGSGNFAEDRERASEAGKKGGQHSGGNFKNDPQRASEAGKKGGKSSHGKSDN     57
PAO1_PA2146        1  MAQHQGGKGNFAEDPKRASEAGKKGGQASGGNFKNDPQRASEAGKKGGQRSHGGN       55
PAO1_PA2190        1  MTDKRQGMST--------SEAGQKGGAA                                  20
                  21        TSRSHGKEFYQEIGHKGGQASGGNFANDPQRAAEAGRKGGQQSGGN          66
                  67          FANDPERAAEAGRKGGQQSGGNFANDREKASEAGRKGGQHSHGGGRSS      114
                                       * ***  ***** **   * *** **** * *
```

Supplementary Figure 6. Phylogenetic relationships of PA2146 homologs.

(A) A Bayesian consensus tree was inferred for aligned nucleotide data using MrBayes3.2.2 software. The analysis was run for three million generations with a HKY nucleotide substitution model, with rates estimated separately for each codon position. Branch support is indicated by posterior probability values inferred from 1000 sampled trees. Nucleotide BLAST identified PA2146 homologs (>75% identity) in ten genera of ɣ-Proteobacteria including *Mixta* (Erwiniaceae), 8 genera of Enterobacteriaceae (*Cedecea, Citrobacter, Enterobacter, Escherichia* (E)*, Klebsiella* (Kleb), *Leclercia, Lelliottia, Shigella*), and 17 species of *Pseudomonas* (Ps). Many taxa had two somewhat divergent homologs at separate genomic sites (e.g., *E. coli* ymdF/yciG, *P. aeruginosa* PA01 PA2146/PA2190). In several cases where copies differed substantially in length, they are designated by "short" or "long" after the strain name. Divergent copies in *C. amalonaticus.92*, *E. asburiae.35953*, *C. cloacae.echih7*, *C. cronae.478*, and *Klebsiella pneumoniae* were similar in length, and designated by ".1" or ".2" appended to the strain name.

(B) Amino acid alignment of PA2146 and its *K. pneumonia* and *E. coli* homologs.

(C) Amino acid alignment of PA2146, its inparalog PA2190 and and its *E. coli* homolog YmdF.

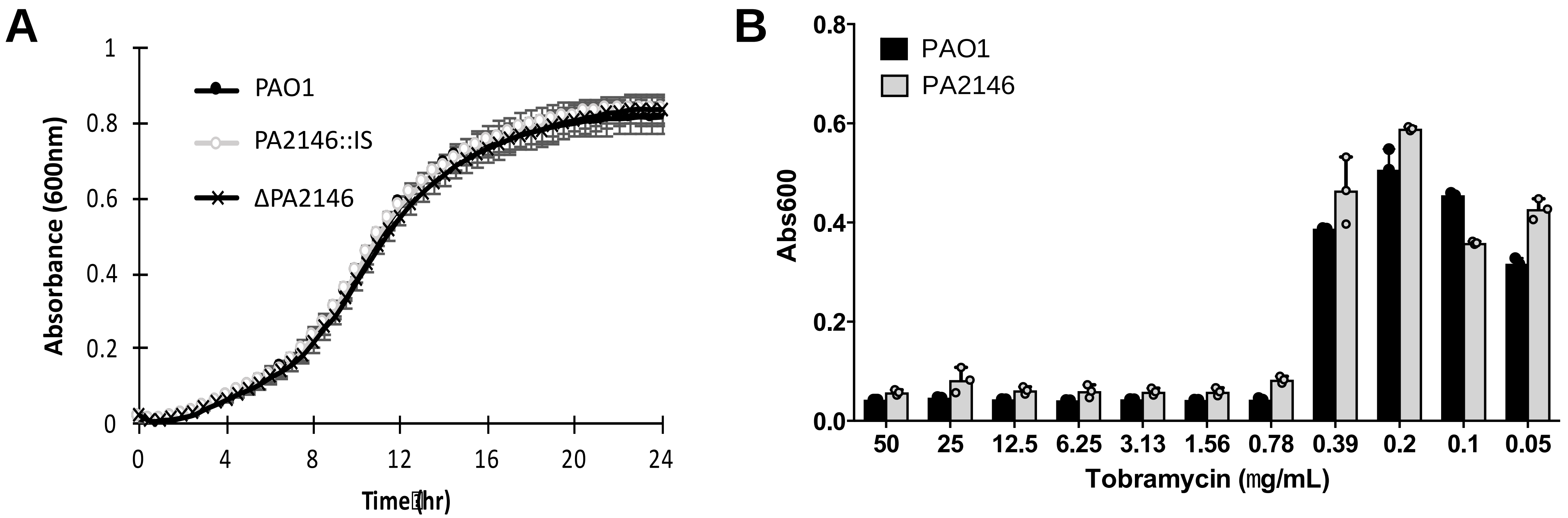

Supplementary Figure 7. Growth and minimum inhibitory concentration (MIC) of *ymdF* homolog and inparalog mutant strains. (A) Growth curves of *P. aeruginosa* PAO1, and mutant strains PA2146::IS and ΔPA2146 were monitored in 96-well plates over the course of 24 h by determining the optical density at 600 nm. All experiments were carried out in triplicate. Error bars indicate standard deviation. (B) Determination of the minimum inhibitory concentration of tobramycin by *P. aeruginosa* PAO1 and the isogenic mutant strain ΔPA2146. Experiment was carried out in triplicate but only representative data are shown.

Supplementary Table 1. Genes encoding hypothetical proteins and demonstrating differential expression during *P. aeruginosa* biofilm growth. Expression of indicated genes in *P. aeruginosa* PAO1 cells grown as biofilms relative to those grown planktonically is shown. Biofilm cells were grown for 3 days under continuous flowing conditions. Planktonic cells were grown to mid exponential phase. Genes with transcript abundance changed ≥2-fold are listed. RNA-seq was performed in triplicate using biological replicates. Relative expression and *p*-values were calculated using EdgeR.

**Locus**	**Relative expression**	***p*-value**
PA2146	19.65452883	8.21E-12
PA3915	16.1114085	9.67E-07
PA2111	10.22905593	9.97E-20
PA0049	9.881226627	5.73E-22
PA3332	9.096295367	1.88E-15
PA2110	9.050246229	4.56E-20
PA1894	8.850054963	1.11E-26
PA3741	7.49095344	8.71E-14
PA0713	7.326463097	3.01E-11
PA5446	7.080141453	2.23E-10
PA5139	7.033422963	9.04E-15
PA3335	6.689385397	2.69E-16
PA1892	6.653644611	3.30E-12
PA0038	6.353209811	4.90E-23
PA2781	6.137149718	1.10E-08
PA3906	6.118124877	2.77E-07
PA3572	6.005666205	3.10E-07
PA2427	5.47498363	7.70E-09
PA5033	5.252432607	1.47E-11
PA2377	5.211378457	2.47E-10
PA0563	4.791978383	3.44E-12
PA4610	4.683877702	1.99E-05
PA3908	4.662835294	5.30E-09
PA2117	4.628281672	9.64E-13
PA1415	4.569545684	3.71E-17
PA0020	4.561229077	1.17E-12
PA0346	4.525423712	4.15E-05
PA4469	4.509815122	1.65E-05
PA1897	4.450382637	2.99E-13
PA2384	4.412948442	7.03E-06
PA2116	4.402175522	4.72E-10
PA2753	4.398147136	1.99E-06
PA0918	4.377089711	9.64E-13
PA3329	4.308720607	1.53E-13
PA2971	4.285131346	5.13E-13
PA2842	4.139548722	3.39E-08
PA3009	4.088427179	7.27E-07
PA1657	4.073540859	2.10E-08
PA2112	4.058846634	2.71E-08
PA2034	4.003583015	5.79E-09
PA2464	3.945474433	1.85E-06
PA2412	3.752394148	3.39E-07
PA0290	3.725782246	4.07E-08

PA4746	3.643967204	8.34E-05
PA4605	3.613943649	5.65E-05
PA3274	3.588979827	0.007490794
PA2184	3.586295105	5.42E-07
PA1095	3.556174102	3.30E-08
PA3907	3.512805236	3.31E-07
PA1761	3.498578103	1.88E-05
PA1096	3.464959374	1.08E-05
PA2436	3.431555247	1.92E-06
PA0078	3.410187887	1.08E-05
PA2134	3.390712475	7.02E-09
PA1658	3.370881274	9.85E-09
PA4639	3.367269927	4.33E-05
PA0952	3.336619649	0.000463759
PA5494	3.3287737	3.07E-07
PA0856	3.317431476	7.54E-07
PA2171	3.311658447	1.74E-09
PA5475	3.30438859	4.03E-07
PA2033	3.267921757	5.21E-06
PA0391	3.265198419	5.62E-08
PA5130	3.250489325	0.000217888
PA4578	3.23749588	6.32E-07
PA0968	3.227969155	1.27E-08
PA4704	3.213201542	4.47E-06
PA0914	3.19078236	0.030347969
PA3922	3.189190819	9.07E-06
PA0084	3.186055519	7.37E-08
PA5108	3.157064612	0.000277854
PA2170	3.138394093	0.113060902
PA3309	3.129093634	7.77E-07
PA2021	3.086047802	0.025961791
PA2017	3.083734677	1.92E-05
PA2365	3.07501575	3.13E-05
PA0572	3.032359452	2.19E-07
PA0868	3.027554033	0.001184823
PA0526	2.999860303	0.024426992
PA0850	2.975547334	0.003545204
PA4570	2.957558725	0.001501562
PA2759	2.898794538	0.001334665
PA2423	2.88077069	9.79E-07
PA2780	2.876653659	0.000212061
PA3923	2.872698578	1.46E-05
PA3836	2.867038037	2.27E-08
PA2764	2.863322412	3.20E-05
PA4939	2.857480761	5.84E-05
PA3986	2.825956517	0.000261925
PA2883	2.823798778	0.000342535
PA2982	2.807005608	1.78E-05
PA4852	2.804827432	0.000127722
PA2366	2.803039409	0.000344635
PA2621	2.799015133	6.27E-05
PA2169	2.796531688	0.001429359
PA1414	2.796198236	0.006326729

PA4835	2.792380597	0.030260268
PA4471	2.777152912	0.000628753
PA1656	2.732766506	2.09E-08
PA1789	2.73211263	2.72E-07
PA0429	2.727911883	4.35E-06
PA3716	2.717067566	8.21E-08
PA2800	2.705709438	5.28E-06
PA5533	2.698737863	9.16E-06
PA4792	2.693244353	0.000133433
PA1896	2.69125656	4.06E-06
PA2137	2.680510475	0.00096834
PA3325	2.679213722	5.68E-07
PA0974	2.676122897	8.25E-08
PA3091	2.673713792	7.55E-05
PA2864	2.666700418	3.05E-05
PA4523	2.622390981	8.28E-05
PA5137	2.612968906	4.01E-05
PA3806	2.608082272	6.58E-08
PA5232	2.60435283	2.23E-05
PA2754	2.588874899	0.000323436
PA3413	2.585803091	0.027031096
PA0050	2.577848126	0.057837736
PA5481	2.577534663	1.19E-06
PA3696	2.568443178	0.001468114
PA5109	2.563570963	1.56E-05
PA3224	2.562655632	7.81E-05
PA1639	2.547761359	0.008302734
PA1714	2.544914961	0.000304421
PA4017	2.527597472	2.00E-05
PA1934	2.512790577	0.002295989
PA3580	2.50546928	0.003417846
PA3854	2.497167912	0.006140684
PA0529	2.470094788	2.69E-05
PA5028	2.464820745	1.53E-06
PA3904	2.46276978	0.005754198
PA5378	2.450215196	1.38E-05
PA0319	2.443827368	4.07E-05
PA3046	2.436122056	0.000168814
PA4638	2.433574117	0.044288727
PA1198	2.412551367	6.17E-05
PA0083	2.406246138	0.000125945
PA3286	2.401778367	1.86E-06
PA5138	2.396954273	7.25E-05
PA0695	2.388305489	0.00372927
PA3726	2.38732661	0.00338187
PA4739	2.374681799	7.48E-06
PA0377	2.373792674	0.012394032
PA3533	2.336962709	3.26E-05
PA5314	2.306532486	0.000695498
PA1618	2.286944945	0.001051463
PA5352	2.277968398	0.036496928
PA3010	2.268471245	6.75E-05
PA3283	2.262247939	0.002748856

PA0566	2.26027147	0.004221369
PA0071	2.258983406	1.60E-05
PA0867	2.253983805	0.00314485
PA2159	2.248232684	0.007823909
PA2190	2.24545104	0.014291232
PA0663	2.238060383	0.000190251
PA4703	2.232649066	0.000194022
PA1830	2.215782022	0.0003816
PA0653	2.214490491	0.000543608
PA3945	2.212943073	4.12E-06
PA2166	2.205995887	0.000127382
PA3998	2.205133259	0.001875739
PA5103	2.201990826	0.000107601
PA5079	2.185248524	0.001443654
PA0384	2.183370118	0.181648298
PA2950	2.177340908	2.06E-05
PA0095	2.173793045	3.49E-06
PA0086	2.161934594	0.002768341
PA2762	2.157636299	0.012254192
PA2372	2.156000073	0.010010806
PA4632	2.154576553	1.93E-05
PA3261	2.149772453	4.04E-06
PA4738	2.146134805	0.000382816
PA3846	2.142712354	0.000531387
PA2143	2.135952927	0.004448813
PA1012	2.134316975	9.31E-05
PA4460	2.133873843	0.000251728
PA1442	2.131587123	0.00898787
PA0769	2.12991832	0.00025709
PA1906	2.127719257	0.003886953
PA3345	2.12697833	0.000377461
PA3017	2.126138883	0.001719622
PA0060	2.125552031	0.004379213
PA1530	2.123836659	0.008672859
PA4404	2.122911042	0.000216381
PA1398	2.110410315	0.01587459
PA4673	2.10852102	0.002467013
PA4325	2.1080365	0.003501524
PA0354	2.101886108	0.001635953
PA2894	2.090746271	0.014948734
PA3919	2.089061894	0.001153013
PA4963	2.084103623	0.000289974
PA4702	2.083198581	0.019409447
PA3764	2.08073336	0.000130049
PA4780	2.074734518	0.001200706
PA3352	2.072943835	0.011280003
PA4872	2.067851088	0.00431334
PA1191	2.064452648	0.015610274
PA2582	2.052493503	0.000208416
PA5363	2.043582822	0.000491915
PA0737	2.039880592	0.027583394
PA1963	2.039834546	0.045182709
PA1116	2.030109879	0.008835739

PA4116	2.025844759	0.005989097
PA0588	2.023054876	0.000889882
PA4717	2.017932955	0.00015531
PA1689	2.015692985	0.000248029
PA2747	2.011799916	0.000363355
PA2769	2.009302589	0.016665353
PA1604	2.003365416	0.000774987
PA3822	2.000438436	0.002478441
PA3461	0.49916241	0.00297946
PA0345	0.49876329	0.039492669
PA2381	0.498520662	0.101012122
PA4509	0.497898049	0.020256022
PA1943	0.497584771	0.00868925
PA2328	0.497582765	0.004386998
PA2530	0.495479718	0.008652165
PA4788	0.495243589	0.06307937
PA1516	0.495092516	0.143896578
PA3496	0.494780056	0.003649533
PA4155	0.493768391	0.04380706
PA0777	0.492391971	0.193807875
PA5226	0.492369531	0.001974015
PA5566	0.490417121	0.266102002
PA1677	0.489769325	0.067279586
PA1508	0.489554057	0.077686634
PA5532	0.489152411	0.018583464
PA2829	0.488975905	0.042098204
PA0046	0.48851754	0.148618209
PA2225	0.487343673	0.092898065
PA3468	0.486495815	0.003065004
PA1837	0.485830801	0.01619063
PA3088	0.485503901	0.003808536
PA2566	0.484654665	6.26E-06
PA1381	0.481481716	0.16356421
PA3464	0.479708664	0.034769475
PA2245	0.479527064	0.119086254
PA0126	0.478583515	0.008576496
PA2789	0.478577022	0.049995628
PA3371	0.47704109	0.140602544
PA4388	0.472823328	0.023214664
PA1536	0.471856245	0.023262302
PA2852	0.471428791	0.001316834
PA2691	0.471208584	0.011278464
PA0234	0.470702347	0.022546494
PA4816	0.47043208	0.043422049
PA2484	0.467482585	0.003402594
PA3229	0.467151506	0.042134832
PA1768	0.465396093	0.002528072
PA0201	0.465098452	0.147309645
PA0812	0.461862987	0.005275806
PA0145	0.461295199	0.072847235
PA1568	0.459529984	0.061895521
PA3675	0.459144534	0.005038272
PA5433	0.456059992	0.03042373

PA3449	0.455972592	0.10778026
PA2839	0.455469121	0.051372138
PA1680	0.45462584	0.122925342
PA3284	0.454533101	0.038175791
PA1494	0.453993144	0.003658087
PA0734	0.452269079	0.272799904
PA5220	0.452115783	0.000637319
PA0573	0.451583816	0.036695415
PA4705	0.450212586	0.053591899
PA5087	0.449456271	0.00292026
PA0497	0.448985793	0.006928499
PA4359	0.448888638	0.301248296
PA2063	0.447551053	0.00237167
PA0696	0.446837187	0.054287123
PA5402	0.446085774	0.031860635
PA0069	0.445536869	0.055677873
PA1210	0.444517355	0.008223697
PA1450	0.444435047	0.005508734
PA2807	0.443657906	0.032608906
PA5191	0.443376827	0.002919188
PA2699	0.442981572	0.004240284
PA2077	0.442007014	0.025147344
PA3422	0.441738197	0.010155221
PA4177	0.441453131	0.101801925
PA3342	0.441131151	0.000343291
PA1953	0.440019146	0.096556588
PA0749	0.438907672	0.010488817
PA0691	0.438840247	0.11309971
PA4883	0.43824455	0.140393208
PA1215	0.437989948	0.022089032
PA2421	0.437467077	0.114174237
PA3026	0.437363657	0.002952244
PA1259	0.437220622	0.005803521
PA3670	0.436796226	0.001882919
PA3840	0.436481608	0.016260248
PA3500	0.434396664	0.017238754
PA2243	0.43278291	0.038132923
PA3202	0.431683745	0.089334464
PA1539	0.431355876	0.005332329
PA1888	0.429710028	8.66E-05
PA2280	0.429498381	0.042355603
PA1974	0.429427574	0.075784692
PA1501	0.429228118	0.00825961
PA2564	0.429216771	2.62E-06
PA4841	0.428512504	0.015764885
PA5086	0.428478024	0.016351796
PA1558	0.428095197	0.027662126
PA3074	0.426550897	0.007456734
PA4629	0.425985706	0.058193132
PA1936	0.425778249	0.050968932
PA4620	0.42473605	0.070706554
PA1486	0.424425238	0.019995802
PA0907	0.423327583	0.007140614

PA4652	0.422831078	0.000845827
PA2182	0.42277449	0.079696325
PA0174	0.422697331	0.027735612
PA0911	0.422249624	0.017670922
PA3515	0.422072914	0.000299628
PA5284	0.422040676	0.003370755
PA3909	0.42167337	0.004487107
PA2201	0.421272561	0.019097022
PA2457	0.420829803	0.000114511
PA1038	0.420408631	0.005962658
PA0284	0.420367051	0.087819076
PA4010	0.420341129	0.000707012
PA3982	0.420196679	0.001173984
PA3119	0.419577518	0.048375956
PA3240	0.418074063	0.001439378
PA2669	0.416984333	0.0363288
PA4871	0.416666688	0.064713584
PA1024	0.416268689	0.010216997
PA3953	0.413856366	0.064607556
PA0121	0.412247822	0.007834109
PA4617	0.41187352	0.001571642
PA2502	0.411533085	0.000511485
PA2448	0.411462389	4.33E-05
PA0238	0.410619149	0.053642888
PA2693	0.409113841	0.090961077
PA4030	0.408387664	0.006248032
PA3779	0.407921844	0.002176824
PA1954	0.40718185	0.037846801
PA0254	0.405073639	0.001286061
PA0845	0.402880215	0.007600461
PA1216	0.402242848	0.015722832
PA0251	0.402122214	0.002029784
PA2418	0.401942157	0.016086162
PA2607	0.401425585	0.023760396
PA1786	0.39975382	0.024346567
PA3196	0.395893956	0.001133419
PA4063	0.395551473	0.065653791
PA1466	0.394913299	0.027999382
PA1255	0.394904668	0.013541212
PA1331	0.394181157	0.001762374
PA3419	0.393011307	0.000570725
PA2463	0.392904678	0.002549986
PA2548	0.392466917	0.001354217
PA3421	0.389926591	0.005395067
PA2598	0.389163386	0.003879099
PA4800	0.388925018	0.013850228
PA1865	0.388576739	0.000383406
PA2844	0.388532203	0.000658035
PA1957	0.387940942	0.052016606
PA2428	0.387600092	0.129053416
PA3505	0.38583137	0.047495023
PA2490	0.385194955	0.065448067
PA4774	0.385185606	0.101060282

PA0875	0.384792262	2.90E-05
PA1186	0.384354733	0.021978433
PA1219	0.384290137	0.021274681
PA4677	0.384017482	8.63E-06
PA5341	0.383256355	0.026786744
PA0277	0.382891622	0.014323513
PA4377	0.382658624	0.000193537
PA0252	0.380577837	0.121850914
PA1168	0.380259513	0.036575706
PA4993	0.380107507	0.000437159
PA3436	0.380043251	0.012356258
PA2689	0.378855106	0.016020434
PA1211	0.377451488	0.018617482
PA2075	0.377172431	0.000203632
PA5467	0.376997567	0.044380891
PA1348	0.376871867	0.000407401
PA1218	0.376219485	0.001006329
PA0925	0.375984358	0.002471654
PA3510	0.375653052	0.052506655
PA1531	0.375004055	0.051928308
PA1153	0.372206523	0.015516088
PA5306	0.371002448	0.000716131
PA3835	0.370722433	0.009101457
PA2803	0.369911816	0.020864601
PA2309	0.368014621	0.015221887
PA4349	0.367871169	4.35E-05
PA2218	0.365684357	0.001447787
PA0690	0.363833457	5.24E-06
PA3209	0.361863151	0.05450738
PA4685	0.360572963	0.041493669
PA3683	0.3600828	0.000680199
PA0574	0.358973616	3.46E-05
PA0339	0.355575122	0.000273684
PA3287	0.354450498	0.011854107
PA2575	0.352820368	0.000489303
PA4485	0.351598061	0.058579954
PA1295	0.351082042	0.010072519
PA0613	0.347669135	0.140380595
PA2282	0.347254849	0.011419964
PA2088	0.345062244	0.001470678
PA0532	0.343124372	0.00434424
PA1514	0.342303356	0.002080909
PA2216	0.340755342	0.006636177
PA2078	0.340455283	0.000488381
PA3577	0.340369396	0.228947689
PA5401	0.337019936	0.01705349
PA2671	0.336560359	0.011863757
PA4653	0.33539071	0.000254082
PA3663	0.333685115	0.000296496
PA2102	0.33309021	0.002190924
PA3140	0.33251926	0.010795827
PA2565	0.332439561	2.40E-05
PA2595	0.331117653	0.011485736

PA0727	0.33090388	0.0002035
PA2057	0.330261507	0.001158531
PA0813	0.32971816	0.00022828
PA4071	0.327972459	0.03688171
PA0058	0.327427679	0.113180371
PA2600	0.327200852	0.015910467
PA2481	0.327004819	0.001582234
PA5088	0.326652113	0.004145596
PA3125	0.325829293	1.39E-05
PA2207	0.323634817	0.001812946
PA5326	0.323366273	0.007614033
PA3066	0.322917822	0.000989818
PA2330	0.322732579	7.37E-06
PA3127	0.32240318	0.00081102
PA0057	0.321604248	0.004606402
PA2498	0.32092101	0.02361994
PA4039	0.320016981	0.000266915
PA1239	0.319560227	0.007757254
PA5120	0.318993018	0.029739243
PA0722	0.317832998	0.014844517
PA3734	0.317337116	7.06E-08
PA2719	0.315022584	0.001306173
PA1467	0.312026668	0.035771537
PA3939	0.311029429	0.009936924
PA0094	0.309993627	0.004646051
PA2136	0.308877243	0.008245928
PA4154	0.307738024	0.024613323
PA2208	0.30652222	0.036756892
PA5534	0.305984039	0.022863806
PA2422	0.305468235	0.007681464
PA4350	0.303278365	0.000658779
PA0054	0.302996129	0.002123097
PA4917	0.302846387	0.004569859
PA4106	0.30226077	0.001082891
PA2590	0.298449638	0.000193075
PA2358	0.297256639	0.008817685
PA4169	0.2971752	0.001679356
PA0269	0.294609834	0.001378867
PA1367	0.294351917	0.000226413
PA2288	0.293810403	0.113481279
PA0222	0.29201639	0.001295673
PA4518	0.290195939	0.006987641
PA2814	0.290028606	0.005483917
PA1969	0.286533399	0.000615856
PA2260	0.285919683	0.014938578
PA0466	0.283462083	0.014003258
PA3566	0.281817412	0.015707184
PA2625	0.281373149	0.034325734
PA2223	0.281118395	4.18E-05
PA3931	0.280931432	0.000313975
PA2296	0.280826979	1.47E-05
PA2602	0.280135033	0.012660609
PA2459	0.277000188	0.000441859

PA2723	0.27648038	0.009893893
PA3446	0.275765962	0.033796334
PA0881	0.273635781	0.00257936
PA2037	0.273324463	2.49E-07
PA4985	0.272203065	0.020210638
PA2750	0.269148739	0.008156244
PA1221	0.269006589	0.000393273
PA2283	0.268416089	0.127838544
PA1935	0.268248251	0.005025775
PA3219	0.26789101	0.061528157
PA5539	0.267660954	0.002760404
PA1607	0.267624815	0.000487005
PA1214	0.266698823	0.00079828
PA3323	0.266452554	0.001556446
PA0726	0.266139588	0.000439329
PA3492	0.2656391	0.000131176
PA0824	0.265322478	0.001014887
PA3513	0.261049274	0.001238024
PA4146	0.259851289	0.000783501
PA3036	0.259032162	0.000508775
PA4220	0.257319769	3.12E-06
PA4182	0.254361354	3.11E-05
PA4384	0.253337514	0.000356433
PA0981	0.253153868	0.000501685
PA4149	0.252942426	0.000850281
PA3488	0.251679812	0.000163254
PA2211	0.250850232	0.000560882
PA3892	0.250467682	8.57E-05
PA1213	0.246186628	0.00039298
PA0912	0.245278169	6.95E-05
PA2222	0.243399265	3.29E-05
PA0274	0.242126296	1.98E-07
PA2429	0.237001877	0.00992926
PA3964	0.234296659	0.000339853
PA3772	0.23380193	2.17E-05
PA2670	0.233182241	7.69E-06
PA5393	0.231213272	0.000140662
PA1412	0.229744996	0.000905255
PA2198	0.228261702	0.006216355
PA2847	0.227165119	0.002963106
PA3868	0.226980695	5.23E-07
PA1026	0.226623994	0.000134994
PA2145	0.226092059	5.73E-05
PA0692	0.225659514	6.83E-08
PA5156	0.223506727	2.65E-05
PA2599	0.222799293	0.000185742
PA2036	0.222141113	0.000499236
PA0270	0.21792387	2.14E-08
PA5196	0.217719932	2.07E-05
PA3412	0.217110772	0.006349196
PA0435	0.216710633	1.06E-05
PA1566	0.216706554	4.65E-05
PA2074	0.213200175	0.000243933

PA4122	0.212909824	0.000411843
PA3445	0.211880181	0.005709767
PA1018	0.211653451	0.001335908
PA1270	0.211558705	6.64E-06
PA1268	0.207330989	0.0007569
PA1488	0.203562748	6.60E-05
PA4836	0.20215242	0.000400299
PA4364	0.201616889	0.002744466
PA1942	0.201401776	0.004549478
PA2336	0.199754042	0.000312313
PA1835	0.199514009	0.00094139
PA0670	0.198946866	0.009419531
PA2228	0.198343264	0.000160766
PA4510	0.197520211	1.88E-06
PA2486	0.197275814	0.002484321
PA0146	0.19506345	0.000594712
PA2048	0.194808204	0.000161643
PA2226	0.193944426	4.30E-07
PA2168	0.19113081	8.07E-06
PA3784	0.189037313	3.36E-11
PA4820	0.188721784	0.007042133
PA5543	0.185051952	0.00084694
PA3592	0.182471533	0.000128606
PA1952	0.182202772	0.000807683
PA0014	0.181195605	0.003027184
PA4103	0.180854921	0.000802096
PA1468	0.180593791	0.001239183
PA4623	0.179941468	0.001740424
PA3519	0.179114075	3.51E-05
PA3518	0.178457629	2.95E-05
PA1220	0.176408371	1.35E-06
PA2090	0.17594728	0.000169652
PA4830	0.174939016	0.001078585
PA3785	0.173713857	9.88E-08
PA0673	0.17099453	0.002564714
PA0980	0.170457872	0.001212493
PA4105	0.169198265	8.20E-05
PA2031	0.163507464	2.80E-07
PA2209	0.161752053	3.90E-05
PA4298	0.161693314	0.000325258
PA2440	0.160859241	1.46E-05
PA0271	0.160772866	3.11E-06
PA2224	0.160201407	2.92E-08
PA5392	0.157979364	0.000866829
PA4188	0.156223982	0.000316519
PA2293	0.155426493	0.001708814
PA1267	0.155117252	0.000786889
PA3205	0.153760918	5.93E-05
PA3783	0.151071676	8.16E-16
PA2935	0.148221433	0.000162636
PA1152	0.147307728	1.11E-05
PA2596	0.144897355	9.25E-06
PA1913	0.138797501	7.05E-07

PA4802	0.136812287	0.000925785
PA4823	0.136116645	0.026347599
PA2285	0.131352907	0.029541554
PA1346	0.125126242	8.15E-07
PA1149	0.116825224	0.000499013
PA4141	0.114650605	1.27E-12
PA1362	0.114175872	0.0030489
PA4170	0.112275561	2.41E-08
PA5391	0.110387149	0.000630033
PA4099	0.109856272	3.95E-08
PA5404	0.108359475	0.001267204
PA4824	0.106824666	0.018043544
PA5115	0.073391638	1.86E-06
PA0671	0.044614531	1.63E-06

Supplementary Table 2. qPCR-based fold change in transcript abundance of selected genes that were found to be differentially expressed upon biofilm growth by RNA-seq. Transcript abundance of *P. aeruginosa* PAO1 cells grown as biofilms relative to those grown planktonically is shown. Biofilm cells were grown for 3 days under continuous flowing conditions. Planktonic cells were grown to mid exponential phase. All experiments were done in triplicate.

		Fold-change relative to planktonic samples			
**PA number**	**Gene name**	**qPCR (logFC)**	**RNA-seq (logFC)**	**qPCR**	**RNA-seq**
**PA0048**		1.50478	3.389287	2.837814	10.477965
**PA0200**		-0.88211	0.492276	0.542574	1.406662
**PA0206**		-1.18747	-0.46293	0.439073	0.72551
**PA0236**		-1.17074	-2.93244	0.444195	0.130993
**PA0246**		-0.28419	-0.2482	0.8212	0.841947
**PA0417**		-1.42921	-0.45164	0.371335	0.731213
**PA0452**		2.39513	3.185457	5.260245	9.097418
**PA0509**	*nirN*	0.87998	1.799087	1.84035	3.479999
**PA0701**	*plkR*	-1.00146	-1.11059	0.499494	0.463104
**PA0713**		2.08713	2.873117	4.24902	7.326463
**PA0918**		1.39087	2.129972	2.622368	4.37709
**PA0979**		-1.9	0.560421	0.267943	1.474699
**PA1131**		-0.4996	1.033625	0.707304	2.047162
**PA1177**	*napE*	-0.5728	0.501291	0.672312	1.41548
**PA1414**		0.51563	1.483467	1.429618	2.796198
**PA1415**		0.87746	2.192051	1.837138	4.569546
**PA1435**		-0.82095	-1.38136	0.566069	0.383857
**PA1541**		-1.32679	-2.12082	0.398655	-1.08462
**PA1762**		-1.1381	-0.16466	0.454358	0.892141
**PA1874**		-0.63126	-0.39759	0.645611	0.759128
**PA1875**		-1.36255	0.027711	0.388895	1.019394
**PA1877**		-0.07586	0.071126	0.948778	1.050536
**PA1897**		2.27508	2.153929	4.840245	4.450383
**PA1957**		-1.25362	-1.36609	0.419395	0.387941
**PA2061**		-3.44222	-1.81895	0.092	0.283427
**PA2114**		2.18563	3.446852	4.549254	10.90451
**PA2146**		6.19574	4.29679	73.3	19.65453
**PA2493**	*mexE*	0.39767	1.38657	1.317379	0.471521

		Fold-change relative to planktonic samples			
**PA number**	**Gene name**	**qPCR (logFC)**	**RNA-seq (logFC)**	**qPCR**	**RNA-seq**
**PA2507**	*catA*	1.2108	2.243944	2.31466	4.736903
**PA2513**	*antB*	0.08685	2.056134	1.062049	4.158703
**PA2583**		-1.5841	-1.15511	0.333533	-0.20803
**PA2620**	*clpA*	0.14114	1.10719	1.102776	2.154256
**PA2753**		1.13279	2.136896	2.192824	4.398147
**PA2838**		-4.26074	-1.57023	0.052166	0.336756
**PA3236**	*betX*	1.46493	2.291468	2.760501	4.895539
**PA3289**		-1.0239	0.842591	0.491785	1.793268
**PA3305.1**	*PhrS*	-1.15134	3.485257	0.450208	11.19868
**PA3309**	*uspK*	0.00639	1.645745	1.004439	3.129094
**PA3327**		1.10483	2.083272	2.150735	4.237673
**PA3334**		0.95854	3.772925	1.943342	13.66984
**PA3337**	*rfaD*	0.16765	1.477396	1.123227	2.784457
**PA3360**		-0.88549	-0.78298	0.541303	0.581164
**PA3364**		-1.66005	-2.12054	0.316429	0.229961
**PA3384**	*phnC*	-0.4847	-1.26635	0.714645	0.415711
**PA3531**	*bfrB*	0.51705	3.269757	1.431026	9.644837
**PA3552**	*arnB*	-3.99116	-2.57277	0.062884	0.168081
**PA3557**	*arnE*	-3.92043	-1.24819	0.066044	0.420976
**PA3572**		1.46727	2.586324	2.764982	6.005666
**PA3573**		1.00532	3.397356	2.007389	10.53674
**PA3789**		-0.69148	-1.34494	0.61922	0.393671
**PA3879**	*narL*	0.80762	1.247444	1.750322	2.374205
**PA4112**		-0.92205	0.308867	0.527757	1.238734
**PA4175**	*piv*	2.98109	4.967084	7.895825	31.27818
**PA4219**	*ampO*	1.4617	-1.419	2.754327	0.373972
**PA4221**	*fptA*	0.84696	-2.00136	1.798707	0.249765
**PA4306**	*flp*	-2.03518	-1.37842	0.243977	0.38464
**PA4352**		-1.90992	0.26181	0.266107	1.198982
**PA4500**		0.31346	1.69752	1.242684	3.243429
**PA4542**	*clpB*	-1.20297	0.572669	0.43438	1.487273
**PA4610**		0.31756	2.227703	1.246221	4.683878
**PA4635**		-0.35735	-0.81591	0.780599	0.568052
**PA4773**		-2.4162	-0.9079	0.187349	0.53296
**PA4777**	*pmrB*	-1.66632	-1.13177	0.315055	0.456356
**PA4877**		-0.55783	0.564832	0.679324	1.479216
**PA4913**		1.93868	3.11134	3.833547	8.64185
**PA5095**		-0.87056	-0.13222	0.546935	0.912427
**PA5099**		-0.94592	-0.24854	0.519098	0.841747

		Fold-change relative to planktonic samples			
**PA number**	**Gene name**	**qPCR (logFC)**	**RNA-seq (logFC)**	**qPCR**	**RNA-seq**
**PA5171**	*arcA*	0.39961	2.544284	1.319151	5.833184
**PA5352**		1.98544	1.187748	3.959834	2.277968
**PA5355**	*glcD*	0.35053	2.28679	1.275029	4.879693
**PA5367**		-1.6863	-0.62723	0.310723	0.64742
**PA5368**		-2.36067	-0.59608	0.194701	0.661549
**PA5369**		-1.39955	0.055626	0.379047	1.03931
**PA5410**	*gbcA*	1.80949	1.743771	3.505184	3.349093
**PA5421**	*fdhA*	0.65153	2.798793	1.570833	6.958581
**PA5427**	*adhA*	-0.16823	1.472245	0.889934	2.774534
**PA5446**		1.47021	2.823778	2.770622	7.080141

Supplementary Table 3. RNA-seq comparison.

Genes identified in this study to demonstrate increased transcript abundance upon biofilm growth and to contribute to the biofilm architecture and/or biofilm susceptibility phenotype are compared to available transcriptome studies based on RNA-seq. The study by Thöming et al. [1] compared gene expression by 77 *P. aeruginosa* clinical isolates under biofilm (BF) and planktonic (PL) growth conditions. The 77 clinical isolates were grouped into biofilm cluster A-C based on distinguishable phenotypes. Doetsch et al. [2] compared the transcriptome of *P. aeruginosa* PA14 grown planktonically to stationary phase to biofilms grown in 96-well plate in LB for 48 hours under static conditions. Only significant changes are shown. Turner et al. [3] demonstrates relative gene expression by *P. aeruginosa* PAO1 relative gene expression in burn and chronic wounds as compared to biofilms grown in MOPS-succinate minimal medium. The study by Cornforth et al. [4] compared the transcriptome *of P. aeruginosa* during human infection to that of *P. aeruginosa* in a variety of laboratory conditions.

Significant changes (FDR ≤ 0.05) and log2-fold changes above or below the threshold (log2FC ≥ 2 or log2FC ≤ -2) are displayed in bold.

P-values are given to the right of each data column.

Strain	Biofilm architecture	Susceptibility phenotype (tobramycin)	Susceptibility phenotype (hydrogen peroxide)	Locus ID	Gene	Description	Thöming et al_clinical isolates	all 77 isolates (Biofilm vs Planktonic)		cluster A (Biofilm vs Planktonic)		cluster B (Biofilm vs Planktonic)		cluster C (Biofilm vs Planktonic)	
**PA0048::IS**	Reduced	WT-like	WT-like	PA0048		putative transcriptional regulator	core	-0.0559	1.0000	0.6842	0.1651	-0.1694	0.8615	0.3510	0.5046
**PA0602::IS**	Reduced	Susceptible	WT-like	PA0602		putative binding protein component of ABC transporter	core	-1.0173	1.0000	-0.9431	0.0001	-1.7105	0.0000	-1.0920	0.0000
**PA0918::IS**	Reduced	Susceptible	WT-like	PA0918		cytochrome b561	core	-1.6197	1.0000	-1.9316	0.0000	-1.1948	0.0029	-1.7986	0.0000
**PA2114::IS**	Reduced	Susceptible	WT-like	PA2114		major facilitator transporter	core	-0.9440	1.0000	-1.5506	0.0476	-0.0915	0.9560	-1.0015	0.2015
**PA2146::IS**	Reduced	Susceptible	Susceptible	PA2146		hypothetical protein	core	**2.5546**	0.6072	**2.8423**	0.0302	**4.9074**	0.0049	1.7830	0.1604
**PA2171::IS**	WT-like	Susceptible	WT-like	PA2171		hypothetical protein	core	0.8979	1.0000	0.4921	0.6934	**2.1177**	0.1708	0.4416	0.7319
**PA2184::IS**	WT-like [illegible]	Susceptible	Susceptible	PA2184		hypothetical protein	NA								
**PA2313::IS**	WT-like	Susceptible	WT-like	PA2313		hypothetical protein	core	1.4143	1.0000	**2.0543**	0.0003	-0.1430	0.8970	1.4273	0.0086
**PA3236::IS**	Reduced	Susceptible	WT-like	PA3236		putative glycine betaine-binding protein precursor	core	1.0228	1.0000	1.2118	0.0004	0.3423	0.5008	1.2963	0.0002
**PA3915::IS**	Reduced	WT-like	Susceptible	PA3915		MoaB1	core	-0.0161	1.0000	0.8462	0.1217	-1.7137	0.0111	1.2852	0.0186
**PA4638::IS**	WT-like	WT-like	Susceptible	PA4638			NA								
**PA4909::IS**	Reduced	Susceptible	WT-like	PA4909		ABC transporter ATP-binding protein	core	0.9002	1.0000	1.0042	0.0205	-0.2115	0.7920	0.8721	0.0426
**PA4913::IS**	Reduced	WT-like	WT-like	PA4913		putative binding protein component of ABC transporter	core	0.7185	1.0000	0.2732	0.5651	0.4590	0.4993	0.8963	0.0433
**PA5033::IS**	Reduced	WT-like	WT-like	PA5033		hypothetical protein	core	-0.1341	1.0000	-0.5158	0.2668	-0.4740	0.4950	0.6496	0.1594
**PA5139::IS**	WT-like	WT-like	Susceptible	PA5139		putative ABC-type amino acid transporter	core	0.1941	1.0000	0.2550	0.4938	0.0073	0.9940	0.5985	0.0921
**PA5421::IS**	Reduced	Susceptible	WT-like	PA5421	fdhA	glutathione-independent formaldehyde dehydrogenase	core	1.1804	1.0000	0.9655	0.0493	0.8561	0.1856	1.1444	0.0185

Strain	Dötsch et al_static biofilm: Biofilm vs Planktonic		Turner et al_RNAseq_Acute Burn and Chronic Surgical Wound Infection: Burn wounds vs. Succ-MOPS grown biofilm		Chronic vs. Succ-MOPS grown biofilm		Cornforth et al_human infection: human vs in vitro		CF sputum vs in vitro biofilm		soft tissue vs in vitro biofilm	
**PA0048::IS**			**2.15**	2.2E-01	**4.67**	6.1E-01	-0.91582982	0.0459644	-0.3293492	0.5992953	**-2.71855427**	0.0023505
**PA0602::IS**			0.98	8.0E-01	0.83	9.4E-01	-0.7832609	0.0462042	-1.24323114	0.0143404	-0.34909705	0.6436625
**PA0918::IS**			**22.16**	3.4E-12	**32.97**	6.2E-09	-0.66395001	0.1595058	-1.49288666	0.0119298	-0.02645874	0.9754429
**PA2114::IS**	**2.56**	5.4E-10	1.24	8.8E-01	2.47	4.8E-01	-0.00354688	0.9975081	0.796314739	0.526305	-2.92620498	0.0312061
**PA2146::IS**	**7.12**	3.9E-76	Inf	1.5E-07	**Inf**	1.1E-02	1.51448428	0.2231923	**-2.52721179**	0.1205279	**2.58191812**	0.1814126
**PA2171::IS**	**3.85**	2.3E-19	**9.59**	8.4E-03	**20.11**	1.4E-01						
**PA2184::IS**			**4.90**	8.6E-02	**22.09**	5.8E-01	-1.32321003	0.6101111	-0.5981882	0.8721729	**-3.47379304**	0.4119885
**PA2313::IS**			**3.02**	2.0E-01	**3.45**	4.4E-01						
**PA3236::IS**	**2.17**	9.7E-06	1.09	8.2E-01	1.70	4.4E-01	0.21113113	0.6259928	0.528184234	0.3230415	-0.3331778	0.6798647
**PA3915::IS**			**5.98**	3.3E-01	**4.35**	1.7E-01	0.81514209	0.2192931	**-2.22611359**	0.0518482	-0.30167959	0.8629414
**PA4638::IS**			0.78	5.2E-01	0.52	1.0E+00	-1.83319625	7.83E-05	-1.62957001	0.0082748	-2.13821631	0.0056992
**PA4909::IS**	**2.72**	2.7E-06	1.29	6.8E-01	**3.35**	5.3E-01	-0.34060103	0.5770413	-1.68179209	0.0255699	0.584586323	0.562952
**PA4913::IS**			**2.85**	6.4E-03	**2.27**	2.9E-01	-1.87546903	0.0007832	-1.72453707	0.0207281	**-2.12404903**	0.0236969
**PA5033::IS**			**5.96**	8.0E-03	**4.64**	1.3E-01	0.03857455	0.924078	-0.61633805	0.1773933	0.647353102	0.2528762
**PA5139::IS**			1.03	9.1E-01	0.46	8.8E-02	-0.86329939	0.0627365	**-2.3111858**	6.04E-05	-0.02377216	0.9768123
**PA5421::IS**	**2.80**	6.8E-08	**3.08**	3.5E-03	0.81	7.9E-01	0.045383266	0.9393919	-0.49338903	0.5041513	0.519198813	0.5847745

Supplementary Table 4. Strains and plasmids used in this study.

Strains/Plasmids	Relevant genotype or description	Source
**Strains**		
***Escherichia coli* laboratory strains**		
DH5α	*F*$^-$ *φ80lacZΔM15 Δ(lacZYA-argF)U169 recA1 endA1 hsdR17(rk*$^-$*, mk*$^+$*) phoA supE44 thi-1 gyrA96 relA1 tonA*	Invitrogen corporation
DHM1	cya-854 recA1 gyrA96 (NaI) thi1 hsdR17 spoT1 rfbD1 glnV44(AS)	[5]
BW25113	Wild-type strain *Δ(araD-araB)567 ΔlacZ4787*(::rrnB-3) *λ*$^-$ *rph-1 Δ(rhaD-rhaB)568 hsdR514*	BL Wanner
ΔymdF$_{Ec}$	*ΔymdF749::kan* in BW25113	[6]
***Pseudomonas aeruginosa* laboratory strains**		
PAO1	Wild-type strain PAO1	B.H. Holloway
ΔsagS	Allelic gene replacement of *sagS* (PA2824) in PAO1	[7]
PA2146::IS	PAO1 transposon mutant in PA2146; Tet^R	[8,9]
ΔPA2146	Allelic gene replacement of PA2146 in PAO1	This study
ΔrsmA	Allelic gene replacement of *rsmA* in PAO1	[10]
PAO1::*lux*	PAO1 control stain with promoterless *lux*	This study
PAO1::P$_{PA2146}$513 -*lux*	*PA2146* promoter *lux* fusion in PAO1	This study
PA0048::IS	PAO1 transposon mutant in PA0048; Tet^R	[8,9]
PA0452::IS	PAO1 transposon mutant in PA0452; Tet^R	[8,9]
PA0602::IS	PAO1 transposon mutant in PA0602; Tet^R	[8,9]
PA0918::IS	PAO1 transposon mutant in PA0918; Tet^R	[8,9]
PA2114::IS	PAO1 transposon mutant in PA2114; Tet^R	[8,9]
PA2116::IS	PAO1 transposon mutant in PA2116; Tet^R	[8,9]
PA2134::IS	PAO1 transposon mutant in PA2134; Tet^R	[8,9]
PA2146::IS	PAO1 transposon mutant in PA2146; Tet^R	[8,9]
PA2171::IS	PAO1 transposon mutant in PA2171; Tet^R	[8,9]
PA2184::IS	PAO1 transposon mutant in PA2184; Tet^R	[8,9]
PA2313::IS	PAO1 transposon mutant in PA2313; Tet^R	[8,9]

	PA2377::IS	PAO1 transposon mutant in PA2377; Tet^R	[8,9]
	PA2508::IS	PAO1 transposon mutant in *catC*/PA2508; Tet^R	[8,9]
	PA2531::IS	PAO1 transposon mutant in PA2531; Tet^R	[8,9]
	PA3236::IS	PAO1 transposon mutant in *betX*/PA3236; Tet^R	[8,9]
	PA3531::IS	PAO1 transposon mutant in *bfrBPA3531*; Tet^R	[8,9]
	PA3572::IS	PAO1 transposon mutant in PA3572; Tet^R	[8,9]
	PA3573::IS	PAO1 transposon mutant in PA3573; Tet^R	[8,9]
	PA3914::IS	PAO1 transposon mutant in *moeA*/PA3914; Tet^R	[8,9]
	PA3915::IS	PAO1 transposon mutant in *moaB1PA3915*; Tet^R	[8,9]
	PA4638::IS	PAO1 transposon mutant in PA4638; Tet^R	[8,9]
	PA4909::IS	PAO1 transposon mutant in PA4909; Tet^R	[8,9]
	PA4913::IS	PAO1 transposon mutant in PA4913; Tet^R	[8,9]
	PA5033::IS	PAO1 transposon mutant in PA5033; Tet^R	[8,9]
	PA5139::IS	PAO1 transposon mutant in PA5139; Tet^R	[8,9]
	PA5421::IS	PAO1 transposon mutant in *fdhA*/PA5421; Tet^R	[8,9]
***Pseudomonas aeruginosa* clinical isolates**			
	Sinusitis 1	Clinical *P. aeruginosa* strain isolated from patient with refractor rhinosinusitis	[11]
	Sinusitis 2	Clinical *P. aeruginosa* strain isolated from patient with refractor rhinosinusitis	[11]
	Burn wound	PA215; *P. aeruginosa* isolated from a chronic wound debridement samples from patients at Southwest Regional Wound Clinic (Lubbuk, TX)	[12]
	Urinary tract	*P. aeruginosa* strain isolated from urine at Bozeman Deaconess Hospital	J. Garth
	Cystic fibrosis 1	CF1-2; Classic *P. aeruginosa* isolate from newborn diagnosed with CF	[13]
	Cystic fibrosis 2	CF1-8; Rough *P. aeruginosa* isolate from newborn diagnosed with CF	[13]
	Cystic fibrosis 3	CF1-13; Mucoid *P. aeruginosa* isolate from newborn diagnosed with CF	[13]
	Cystic fibrosis 4	A1; *P. aeruginosa* isolate from patient diagnosed with CF	P. Singh
	Cystic fibrosis 5	A2; *P. aeruginosa* isolate from patient diagnosed with CF	P. Singh
***Klebsiella pneumoniae* laboratory strains**			
	MKP103	KPNIH1-derivative parent strain for *K. pneumoniae* transposon mutant library	[14]
	ymdF1_{Kp}::T30	KP04231 from *K. pneumoniae* MKP103 transposon mutant library; KPNIH1_09545-147::T30	[14]

	*ymdF*2$_{Kp}$::T30	KP04419 from *K. pneumoniae* MKP103 transposon mutant library; KPNIH1_10100-187::T30	[14]
	*ymdF*3$_{Kp}$::T30	KP05857 from *K. pneumoniae* MKP103 transposon mutant library; KPNIH1_13915-149::T30	[14]
**Plasmids**			
	pRK2013	Helper plasmid for triparental mating; *mob; tra*; Km^R	[15]
	mini-CTX-*lux*	Integration-proficient vector for single-copy chromosomal *lux* gene fusions, Tet^R	[16]
	CTX-PA2146-*lux*	Mini-CTX-*lux* with 1-513 upstream region of PA2146); Tet^R	This study
	pEX18Gm	Conjugative suicide plasmid for allelic gene replacement; Gm^R	[17]
	pFLP2	Plasmid to enable Flp-FRT-mediated excision of vector backbones; Gm^R	[18]
	pEX18-PA2146	PA2146 (*Pa-ymdF*) gene replacement vector; Gm^R	This study
	pMJT-1	*araC*-P_{BAD} cassette of pJN105 cloned into pUCP18, Amp^R (Carb^R)	[19]
	pMJT-PA2146_V5	C-terminal His$_6$/V5-tagged PA2146 cloned into pMJT1; Amp^R (Carb^R)	This study
	pMJT-PA2146	PA2146(*ymdF*$_{Pa}$) cloned into pMJT1; Amp^R (Carb^R)	This study
	pMJT-*rsmA*	*rsmA* cloned into pMJT-1 at NheI/SacI; Amp^R (Carb^R)	This study
	pMJT-*csrA*	*csrA* cloned into pMJT-1 at NheI/SacI; Amp^R (Carb^R)	This study
	pMJT-*ymdF*$_{Ec}$	*ymdF* cloned into pMJT-1 at NheI/SacI; Amp^R (Carb^R)	This study
	pMJT-*sagS*_HA	C-terminally HA-tagged *sagS* cloned into pMJT-1 at NheI/SacI; Amp^R (Carb^R)	[7]
	pMJT-*ymdF1*$_{Kp}$	KPNIH1_09545 cloned into pMJT-1 at NheI/SacI; Amp^R (Carb^R)	This study
	pMJT-*ymdF2*$_{Kp}$	KPNIH1_10100 cloned into pMJT-1 at NheI/SacI; Amp^R (Carb^R)	This study
	pMJT-*ymdF2*$_{Kp}$	KPNIH1_13915 cloned into pMJT-1 at NheI/SacI; Amp^R (Carb^R)	This study
	pJN105	Arabinose-inducible gene expression vector; pBRR-1 MCS; *araC*-P_{BAD}, Gm^R	[20]
	pJN-PA2184_HA	C-terminal HA-tagged PA2146 cloned into pJON105; Gm^R	This study
	pKT25	BACTH vector allowing fusion to the C-terminus of the *cyaA* T25 fragment; Kan^R	[5]
	pUT18c	BACTH vector allowing fusion to the C-terminus of the *cyaA* T18 fragment; Amp^R	[5]
	pKT25-*torR*	D2 domain of TorR cloned into pKT25; Kan^R	[21]
	pUT18c-*torS*	Hpt domain of TorS cloned into pUT18c; Amp^R	[21]
	pUT18c-PA2184	Full length PA2184 cloned into pUT18c; Amp^R	This study
	pKT25-PA2146	Full length PA2146 cloned into into pKT25; Kan^R	This study
	pCdrA::*gfp*(ASV)	pUCP22Not-P_{cdrA}-RBS-CDS-RNase III-*gfp*(ASV)-T_0-T_1, Amp^R, Gm^R	[22]

Supplementary Table 5. Oligonucleotides used in this study

NAME	SEQUENCE[1,2]
**Cloning into pMJT-1 or pJN105**	
**PA2146_NheI_for**	GCGCGCGCgctagcATGGCACAGCATCAAGGTGG
**PA2146_SacI_rev**	GCGCGCGCgagctcCACTTCACTGCGCGGTTATC
**PA2146_V5_SacI_rev**	GCGCGCGCgagctcTCAcgtagaatcgagaccgaggagagggttagggataggcttaccGTTCCCGCCGTGGCTGCG
PA2184_HA_EcoRI_for	GCGCGCGCgaattcATGACGAGCAAACAGGAAAACC
PA2184_HA_XbaI_rev	GCGCGCGCtctagaTCAagcgtagtctgggacgtcgtatgggtaTCGCTTGGCCTCGGTGTCCG
**Ec_yciG_NheI_for**	GCGCGCGCgctagcATGGCCGAACATCGTGGTGG
**Ec_yciG_SacI_rev**	GCGCGCGCgagctcTCAGGATTTGCCTGATTTATTACC
**Ec_ymdF_NheI_for**	GCGCGCGCgctagcATGGCAAACCATCGAGGCGG
**Ec_ymdF_SacI_rev**	GCGCGCGCgagctcCTAGTTGTCGCTTTTGCCGTGA
**rsmA_NheI_for**	GCGCGCGCgctagcATGCTGATTCTGACTCGTCGG
**rsmA_SacI_rev**	GCGCGCGCgagctcTTAATGGTTTGGCTCTTGATCTTTC
**EC_csrA_NheI_for**	GCGCGCGCgctagcATGCTGATTCTGACTCGTCGA
**Ec_csrA_SacI_rev**	GCGCGCGCgagctcTTAGTAACTGGACTGCTGGGA
**KPNIH1_09545_NheI_for**	GCGCGCGCgctagcATGGCAAACCATCGTGGCGG
**KPNIH1_09545_SacI_rev**	GCGCGCGCgagctcACTTTCACGACTACCATGACTG
**KPNIH1_10100_NheI_for**	GCGCGCGCgctagcATGGCAGAGCATCGTGGTGG
**KPNIH1_10100_SacI_rev**	GCGCGCGCgagctcGGAATTATCGGACTTGCGGC
**KPNIH1_13915_NheI_for**	GCGCGCGCgctagcATGGCAGAGCATAGAGGCGG
**KPNIH1_13915_SacI_rev**	GCGCGCGCgagctcGCTGTCACCGGATTTACGGC
**Cloning PA2146 allelic replacement construct into pEX18Gm**	
**PA2146_EcoRI_For1**	GCGCGCGCgaattcGCTTTCATCTGACGCAATTCG
**PA2146_BamHI_Rev1**	GCGCGCGCggatccCTCCGAATAATCTCTTCAGAAC
**PA2146_BamHI_For2**	GCGCGCGCggatccCGGCAACTTCAAGAACGATCC
**PA2146_HindIII_Rev2**	GCGCGCGCaagcttCTCTCCTCATCCTGGGCATG
**PA2146_chk_for2**	CACTGTTTTCCATGGCGAGTTCC
**PA2146_chk_rev2**	CTGCACGGTGGAGAAACGCAC
**Cloning PA2146 reporter constructs into mini-CTX-*lux***	
**PA2146(-1)_rev_BamHI**	GCGCGCggatccTTCTATTTCCTCCGAATAATCTCTTC
**PA2146(-125)_for_EcoRI**	GCGCGCgaattcGCGCTTCGCTTTCATTCGCTG
**PA2146(-251)_for_EcoRI**	GCGCGCgaattcCATGGTGCTCCTCCGAGATG
**PA2146(-513)_for_EcoRI**	GCGCGCgaattcGTCACTGTTTTCCATGGCGAG
**Cloning for Bacterial-2-hybrid assay**	
**PA2146_KT25_XbaI_for**	GCGCGCGCtctagaGATGGCACAGCATCAAGGTGG
**PA2146_KT25_ SmaI_rev**	GCGCGCGCcccgggTCAGTTCCCGCCGTGGCTG
PA2184_T18c_XbaI_for	GCGCGCGCctcagaGATGACGAGCAAACAGGAAAACC

**PA2184_T18c_SacI_rev**	GCGCGCGCgagctcTCATCGCTTGGCCTCGGTGTCC
**pKT25_MCS_for**	CGACATGTTCGCCATTATGC
**pKT25_MCS_rev**	CGGGCCTCTTCGCTATTAC
**pUT18c_MCS_for**	GCCTGTTCGACGATGGG
**pUT18c_MCS_rev**	CTCTGACACATGCAGCTC
***E. coli* qRT-PCR primers**	
**Ec_ymdF_RT_for**	GCAGAAGACCGCGAAAGAGC
**Ec_ymdF_RT_rev**	CGCTTTTGCCGTGACTGCTC
**Ec_mreB_RT_for**	GTCCATTGACCTGGGTACTGC
**Ec_mreB_RT_rev**	CATCTGCTTCGCGTCATGACC
**Ec_csrA_RT_for**	GAGTTGGTGAGACCCTCATG
**Ec_csrA_RT_rev**	GATACGCTGGTAGATCTCTTC
***P. aeruginosa* qRT-PCR**	
**PA2146_RT_for1**	CAGCATCAAGGTGGTAAAGG
**PA2146_RT_rev2**	CTGCGCTGGCCACCTTTC
**mreB-for**	CTGTCGATCGACCTGGG
**mreB-rev**	CAGCCATCGGCTCTTCG
**PA_rsmA_RT_for**	GGTCGGAGAGACCCTGATG
**PA_rsmA_RT_rev**	GTGTACGGCGACTTCCTTC
***K. pneumoniae* qRT-PCR primers**	
**KPNIH1_09545_RT_for**	GCTGAAGACCGTGAAAGAGC
**KPNIH1_09545_RT_rev**	CTTTCACGACTACCATGACTG
**KPNIH1_10100_RT_for**	GAGCATCGTGGTGGTTCCG
**KPNIH1_10100_RT_rev**	GAATTATCGGACTTGCGGCC
**KPNIH1_13915_RT_for**	GAGCATAGAGGCGGTTCAGG
**KPNIH1_13915_RT_rev**	CCATGACTGTTCTTACCACCC
**Kp_rpoB_RT_for**	GTGTTATCGTTTCTCAGCTGC
**Kp_rpoB_RT_rev**	GTCAAGGATCTGCTCAGTGG

[1] Lowercase letters indicate restriction sites

[2] Underlined lowercase letters indicate the sequence encoding the V5 epitope tag

REFERENCES

1 Thöming, J. G. *et al.* Parallel evolutionary paths to produce more than one *Pseudomonas aeruginosa* biofilm phenotype. *npj Biofilms and Microbiomes* **6**, 2, doi:10.1038/s41522-019-0113-6 (2020).

2 Dötsch, A. *et al.* The *Pseudomonas aeruginosa* transcriptome in planktonic cultures and static biofilms using RNA sequencing. *PloS one* **7**, e31092 (2012).

3 Turner, K. H., Everett, J., Trivedi, U., Rumbaugh, K. P. & Whiteley, M. Requirements for *Pseudomonas aeruginosa* acute burn and chronic surgical wound infection. *PLoS genetics* **10**, e1004518 (2014).

4 Cornforth, D. M. *et al. Pseudomonas aeruginosa* transcriptome during human infection. *Proceedings of the National Academy of Sciences* **115**, E5125-E5134, doi:10.1073/pnas.1717525115 (2018).

5 Karimova, G., Dautin, N. & Ladant, D. Interaction network among *Escherichia coli* membrane proteins involved in cell division as revealed by bacterial two-hybrid analysis. *Journal of Bacteriology* **187**, 2233-2243, doi:10.1128/jb.187.7.2233-2243.2005 (2005).

6 Baba, T. *et al.* Construction of *Escherichia coli* K12 in-frame, single-gene knockout mutants: the Keio collection. *Molecular Systems Biology* **2**, 2006.0008, doi:10.1038/msb4100050 (2006).

7 Petrova, O. E. & Sauer, K. SagS contributes to the motile-sessile switch and acts in concert with BfiSR to enable *Pseudomonas aeruginosa* biofilm formation *Journal of Bacteriology* **193**, 6614-6628 (2011).

8 Jacobs, M. A. *et al.* Comprehensive transposon mutant library of *Pseudomonas aeruginosa*. *Proceedings of the National Academy of Sciences* **100**, 14339-14344, doi:10.1073/pnas.2036282100 (2003).

9 Held, K., Ramage, E., Jacobs, M., Gallagher, L. & Manoil, C. Sequence-verified two-allele transposon mutant library for *Pseudomonas aeruginosa* PAO1. *Journal of Bacteriology* **194**, 6387-6389 (2012).

10 Kay, E. *et al.* Two GacA-dependent small RNAs modulate the quorum-sensing response in *Pseudomonas aeruginosa*. *Journal of Bacteriology* **188**, 6026-6033 (2006).

11 Desrosiers, M., Myntti, M. & James, G. Methods for removing bacterial biofilms: in vitro study using clinical chronic rhinosinusitis specimens. *American journal of rhinology* **21**, 527-532 (2007).

12 Ammons, M. C. B., Ward, L. S., Fisher, S. T., Wolcott, R. D. & James, G. A. *In vitro* susceptibility of established biofilms composed of a clinical wound isolate of *Pseudomonas aeruginosa* treated with lactoferrin and xylitol. *International journal of antimicrobial agents* **33**, 230-236 (2009).

13 Ogle, J. W., Janda, J. M., Woods, D. E. & Vasil, M. L. Characterization and use of a DNA probe as an epidemiological marker for *Pseudomonas aeruginosa*. *J. Infect. Dis.* **155**, 119-126, doi:10.1093/infdis/155.1.119 (1987).

14 Ramage, B. *et al.* Comprehensive arrayed transposon mutant library of *Klebsiella pneumoniae* outbreak strain KPNIH1. *Journal of Bacteriology* **199**, e00352-00317, doi:10.1128/jb.00352-17 (2017).

15 Figurski, D. H. & Helinski, D. R. Replication of an origin-containing derivative of plasmid RK2 dependent on a plasmid function provided *in trans*. *Proceedings of the National Academy of Sciences* **76**, 1648-1652 (1979).

16 Becher, A. & Schweizer, H. P. Integration-proficient *Pseudomonas aeruginosa* vectors for isolation of single-copy chromosomal *lacZ* and *lux* gene fusions. *Biotechniques* **29**, 948-952 (2000).

17 Hoang, T. T., Karkhoff-Schweizer, R. R., Kutchma, A. J. & Schweizer, H. P. A broad-host-range Flp-FRT recombination system for site-specific excision of chromosomally-located DNA sequences: application for isolation of unmarked *Pseudomonas aeruginosa* mutants. *Gene* **212**, 77-86 (1998).
18 Choi, K. H. & Schweizer, H. P. mini-Tn7 insertion in bacteria with single attTn7 sites: example *Pseudomonas aeruginosa. Nature Protocol* **1**, 153-161 (2006).
19 Kaneko, Y., Thoendel, M., Olakanmi, O., Britigan, B. E. & Singh, P. K. The transition metal gallium disrupts *Pseudomonas aeruginosa* iron metabolism and has antimicrobial and antibiofilm activity. *J. Clin. Invest.* **117**, 877-888 (2007).
20 Newman, J. R. & Fuqua, C. Broad-host-range expression vectors that carry the L-arabinose-inducible Escherichia coli araBAD promoter and the araC regulator. *Gene* **227**, 197-203, doi:10.1016/s0378-1119(98)00601-5 (1999).
21 Kulasekara, H. D. *et al.* A novel two-component system controls the expression of *Pseudomonas aeruginosa* fimbrial *cup* genes. *Molecular Microbiology* **55**, 368-380 (2005).
22 Rybtke, M. T. *et al.* Fluorescence-based reporter for gauging cyclic di-GMP levels in Pseudomonas aeruginosa. *Appl Environ Microbiol* **78**, 5060-5069, doi:10.1128/AEM.00414-12 (2012).

Uncropped blots

Uncropped blot (X-ray film on top of PVDF membrane showing protein marker)

Boxed in area corresponds to blot shown in Figure 4

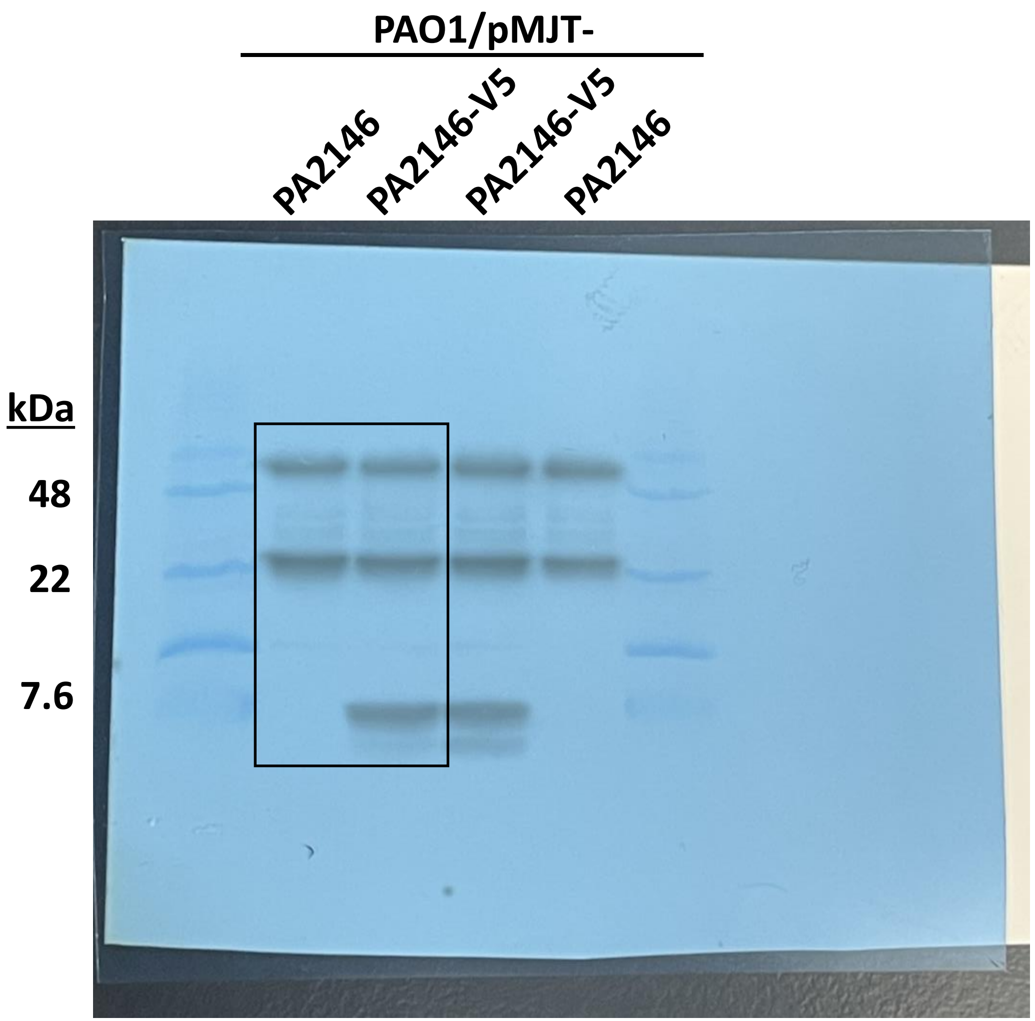

Uncropped blot (X-ray film on top of PVDF membrane showing protein marker)

Boxed in area corresponds to blot shown in Figure 8

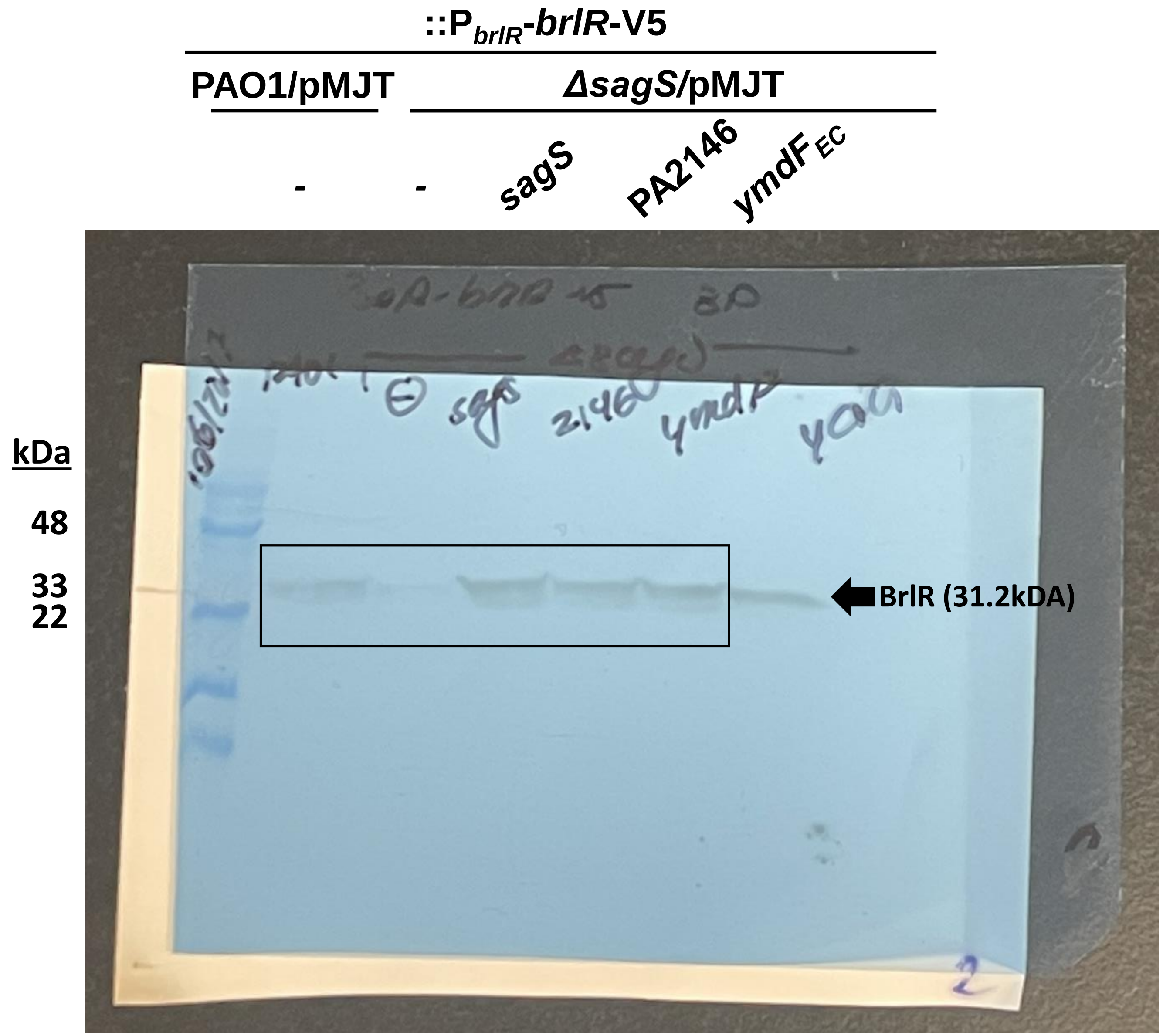

Uncropped blot (X-ray film on top of PVDF membrane showing protein marker)

Boxed in area corresponds to blot shown in Figure 8

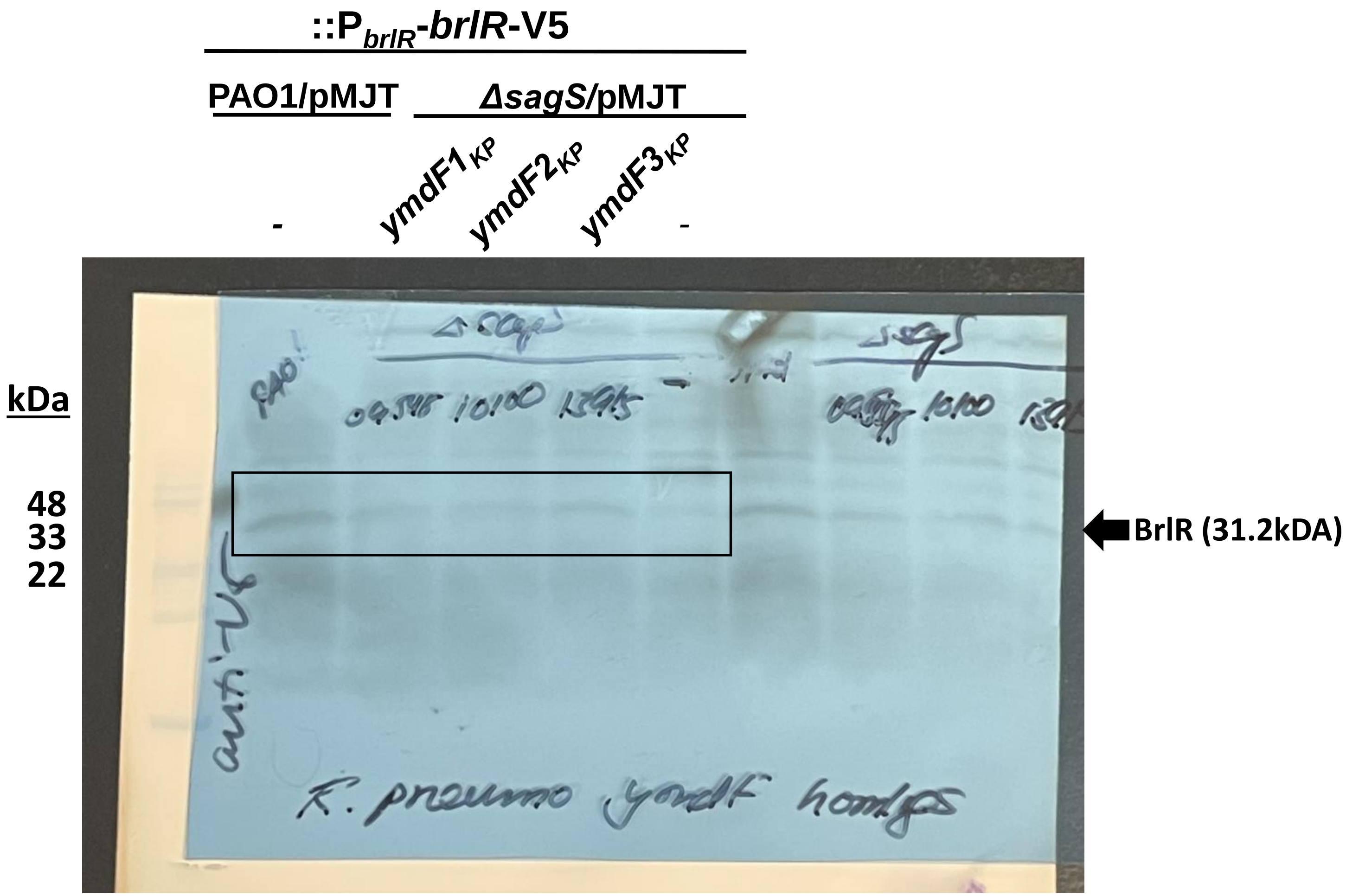

Uncropped blot (X-ray film on top of PVDF membrane showing protein marker)
Boxed in area corresponds to blot shown in Supplementary Figure 5

pMJT-PA2146-V5

pJN105 PA2184-HA PA3915-HA

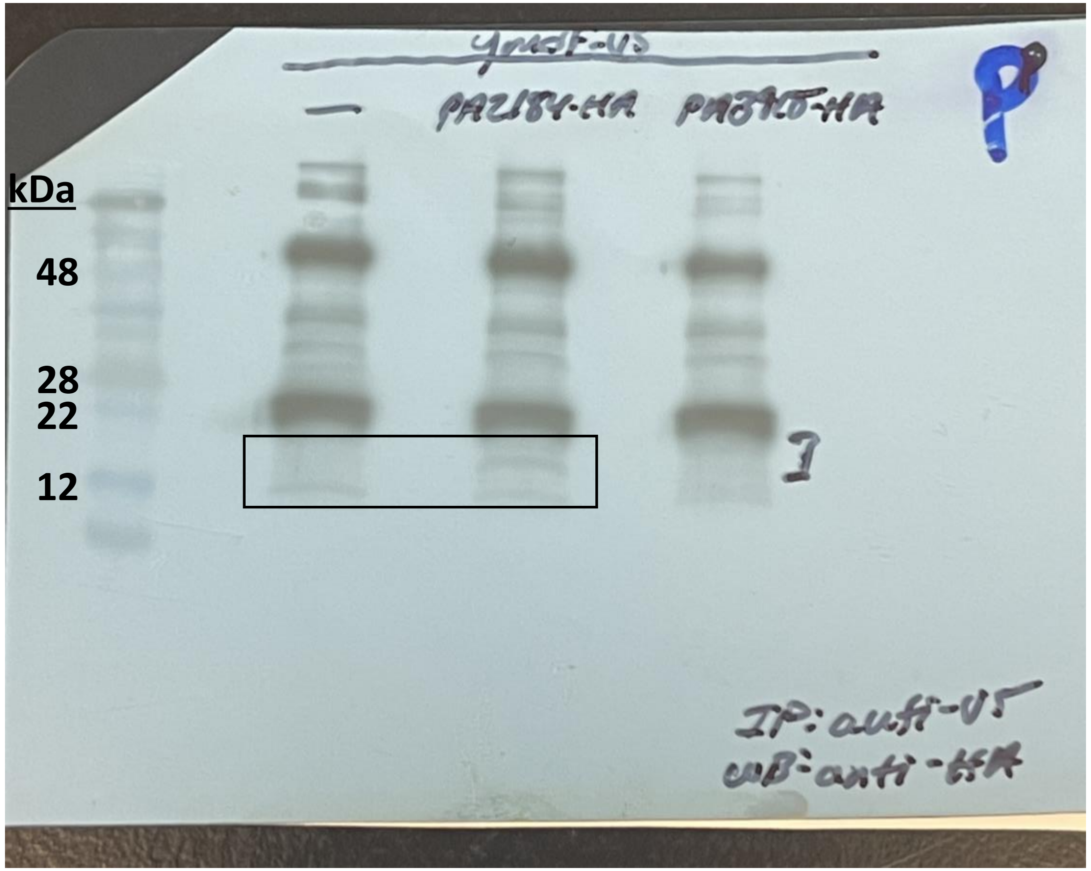

pMJT-PA2146-V5

pJN105 PA2184-HA PA3915-HA

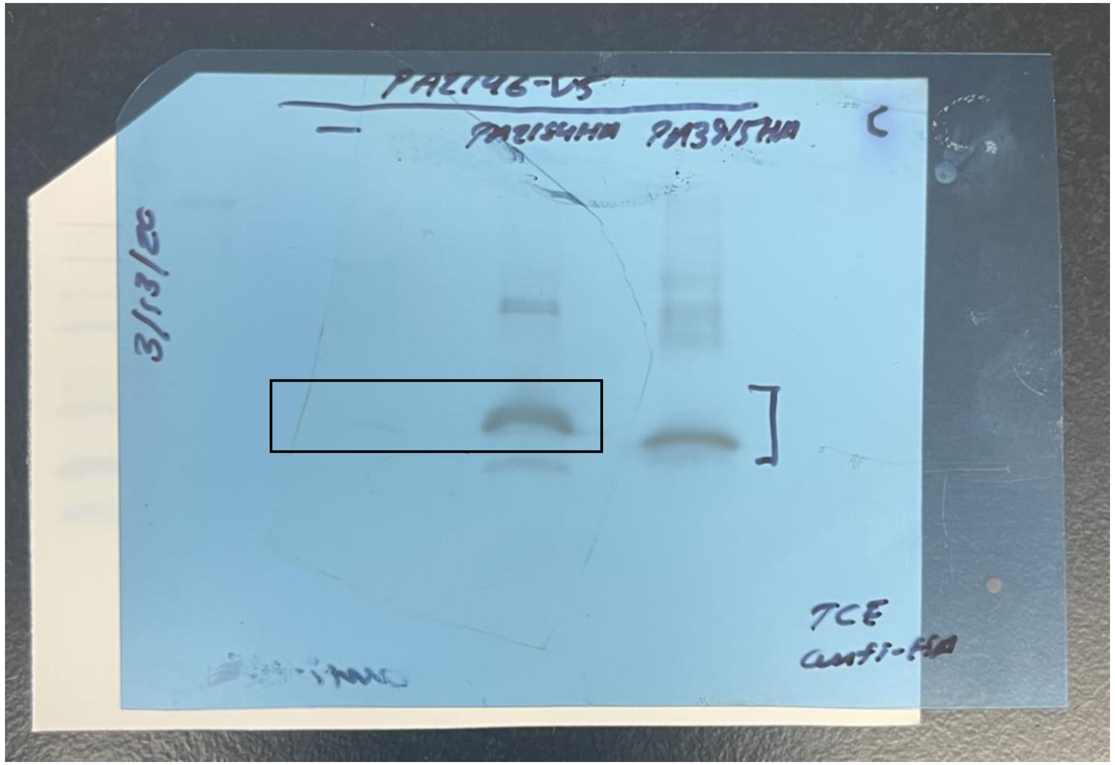

Supplement: Supplementary file 1 — Supplementary material [file 41522_2022_314_MOESM1_ESM.pdf]
